# Using Cross‐Correlated Spin Relaxation to Characterize Backbone Dihedral Angle Distributions of Flexible Protein Segments

**DOI:** 10.1002/cphc.202000789

**Published:** 2020-12-10

**Authors:** Clemens Kauffmann, Anna Zawadzka‐Kazimierczuk, Georg Kontaxis, Robert Konrat

**Affiliations:** ^1^ Department of Structural and Computational Biology Max Perutz Laboratories University of Vienna Vienna Biocenter Campus 5 A-1030 Vienna Austria; ^2^ Biological and Chemical Research Centre Faculty of Chemistry University of Warsaw Żwirki i Wigury 101 02-089 Warsaw Poland

**Keywords:** cross-correlated relaxation, NMR spectroscopy, protein dynamics, protein structures, statistical inference

## Abstract

Crucial to the function of proteins is their existence as conformational ensembles sampling numerous and structurally diverse substates. Despite this widely accepted notion there is still a high demand for meaningful and reliable approaches to characterize protein ensembles in solution. As it is usually conducted in solution, NMR spectroscopy offers unique possibilities to address this challenge. Particularly, cross‐correlated relaxation (CCR) effects have long been established to encode both protein structure and dynamics in a compelling manner. However, this wealth of information often limits their use in practice as structure and dynamics might prove difficult to disentangle. Using a modern Maximum Entropy (MaxEnt) reweighting approach to interpret CCR rates of Ubiquitin, we demonstrate that these uncertainties do not necessarily impair resolving CCR‐encoded structural information. Instead, a suitable balance between complementary CCR experiments and prior information is found to be the most crucial factor in mapping backbone dihedral angle distributions. Experimental and systematic deviations such as oversimplified dynamics appear to be of minor importance. Using Ubiquitin as an example, we demonstrate that CCR rates are capable of characterizing rigid and flexible residues alike, indicating their unharnessed potential in studying disordered proteins.

## Graphical abstract

**Cross‐correlated spin relaxation (CCR)** rates uniquely encode both protein structure and dynamics but their quantitative interpretation is challenging in the presence of conformational heterogeneity. Using a Maximum Entropy approach, we demonstrate that CCR rates are capable of characterizing dihedral angle distributions of both rigid and flexible residues in Ubiquitin.

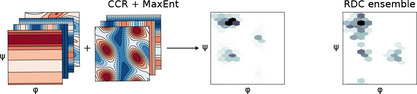

## 1 Introduction

The structure‐function‐paradigm is compelling not only due to its conceptual simplicity. The possibility to describe a protein system by a singular minimum energy configuration greatly reduces the complexity by which the underlying structural ensemble has to be modeled. Most importantly, the assumed absence of conformational averaging allows to derive a singular model structure directly from the experimental data, a key concept of conventional structure calculation methods.^[1]^ However, this simplifying assumption fails in case of pronounced structural flexibility. While arguably inconvenient for structure determination purposes, the ability to sample numerous and structurally diverse substates is crucial for many proteins to function^[2]^ as it allows for important regulatory processes such as allosteric regulation.[^3^, ^4^, ^5^] An extreme case of conformational flexibility is found in so‐called intrinsically disordered proteins (IDPs) that appear unstructured under native conditions. Disordered regions of at least 30 residues in length are estimated to occur in 10–35 % of prokaryotic and 15–45 % of eukaryotic proteins.^[6]^

In order to model and characterize protein systems in their full structural diversity, methods and protocols are required that do not make overly restrictive assumptions about the characteristics of the underlying ensemble. However, reconstructing ensembles from averaged data is generally an ill‐posed problem, i. e. very different ensembles might be compatible with the data at hand, and only a substantial increase in the number of experimental observables can possibly alleviate this problem. NMR spectroscopy offers numerous experimental parameters to study protein conformational ensembles in solution, such as hydrogen exchange rates (HDX), scalar couplings (3J), chemical shifts (CS), residual dipolar couplings (RDCs), nuclear Overhauser effects (NOEs) and paramagentic relaxation enhancements (PREs), allowing to probe (even transient) structural elements as well as their encoded dynamics with atomic resolution.[^7^, ^8^, ^9^]

Here we advocate a less common approach using cross‐correlated relaxation (CCR) mechanisms to probe backbone dihedral angle distributions of proteins in solution. Cross‐correlated relaxation arises from interference effects between the fluctuations of different relaxation mechanisms, typically between two different dipolar (DP) and/or chemical shift anisotropy (CSA) interactions. Any two relaxation mechanisms arising from equal rank tensorial interactions can lead to observable relaxation interference. While, in theory, independent of their respective distance, in practice, this is of course limited by the effective means of coherence transfer involving more remote nuclei. Still, interactions within sequentially adjacent residues are generally accessible, allowing for a wide variety of CCR effects to be probed,[^10^, ^11^] most of which depend on the backbone dihedral angles. While inherently non‐injective, these dependencies have been shown to be complementary, i. e. CCR rates can be analyzed in a combined fashion to resolve their mutual ambiguities assuming a stable fold.[^12^, ^13^] We argue that this complementarity goes further than simply allowing to resolve angular ambiguities in folded proteins.

We have recently demonstrated that CCR rates reveal surprising structural and dynamical propensities in intrinsically disordered proteins (IDPs).^[14]^ However, in the presence of substantial conformational averaging, quantitative interpretation of these rates appears difficult due to their non‐trivial convolution of structural and dynamical contributions. Uncertainties about the dynamics translate into uncertainties about the structure and vice versa. For this reason, CCR rates seem to demonstrate their full potential predominantly in well‐characterized protein systems.[^15^, ^16^, ^17^, ^18^, ^19^, ^20^] Using Ubiquitin as an example, we show that imprecisely modeled protein dynamics do not necessarily obfuscate CCR‐encoded structural information. It is neither necessary to require precise quantitative agreements between model and experiment nor to assume a rigid backbone to reliably characterize backbone dihedral angle distributions. Using a Maximum Entropy (MaxEnt) approach, we find that the complementary nature of CCR rates allows them to compensate for dynamical and geometrical uncertainties even in cases of substantial structural flexibility.

While traditional approaches developed for folded proteins are often ill‐equipped to model heterogeneous structural ensembles, MaxEnt inspired methods have made tremendous progress in the recent past. Particularly promising are MaxEnt *reweighting* approaches, which have matured to well‐founded and efficient protocols over the last few years.[^21^, ^22^, ^23^, ^24^, ^25^] Given a predefined population‐weighted set of conformations, called the *prior*, these methods form an ensemble estimate by reweighting the prior as little as necessary to match the experimental data. This works well in practice as long as all experimentally relevant conformations can safely be specified a priori.^[26]^ While this requirement cannot be met in every case, it does generally apply if the protein ensemble is considered not in its full 3D complexity but in terms of the experimentally relevant variables, such as interatomic distances or backbone dihedral angles. This dimensionality reduction not only allows for simple and extensive definitions of the prior conformations but also tends to increase the relative information content of the experiments. A backbone dihedral angle distribution is more easily defined and restrained than conformational space in 3D. Thus, we consider MaxEnt reweighting as the method of choice to assess the structural information encoded by CCR rates.

We start by deriving an alternative approach to existing MaxEnt heuristics from first principles. Applying the method on Ubiquitin, we find that CCR‐guided MaxEnt reweighting is capable of characterizing rigid and flexible protein backbone regions alike. Most importantly, our results suggest that neither experimental nor systematic errors are the most relevant factors in mapping backbone dihedral angle distributions, but rather a reasonable balance between complementary experiments and prior information. Our findings indicate a surprisingly low sensitivity to experimental uncertainties and oversimplified dynamics, highlighting the potential of CCR rates for the characterization of conformational ensembles of proteins.

## 2 Theory

### 2.1 Cross‐Correlated Relaxation

Cross‐correlated relaxation (CCR) effects result from correlated interferences of simultaneous spin relaxation processes. Using these effects to study protein backbone geometry was first proposed in the late 90s by Reif et al.,^[27]^ who deduced *ψ* from relaxation interference of interresidual $C^{\alpha }$ ‐$H^{\alpha }$ and *N*‐*H* ^*N*^ dipolar vectors. Other interactions probing dihedral angles along the protein backbone were soon proposed,[^28^, ^29^, ^30^, ^31^, ^32^, ^33^, ^34^] for more in‐depth reviews see e. g. Refs. [10, 11, 35].

The angular information is encoded in the spectral density function, which is most commonly modeled under the simplifying assumption of isotropic molecular tumbling with no internal dynamics.[^27^, ^28^] The correlation time is often scaled by a heuristic order parameter^[36]^ to mimic the effects of local motions,[^11^, ^13^] see Refs. [11, 37] for more sophisticated models. Here, we consider the most commonly exploited types of relaxation mechanisms, dipolar (*DP*) and chemical shift anisotropy (*CSA*), under the simplifying assumptions above. Three different combinations can be distinguished,

(1) $$\Gamma_{ab,cd}^{DP,DP}\left(\theta \right)=\;\frac{2}{5}{\left(\frac{\mu_{0}\hbar }{4\pi }\right)}^{2}\;\frac{\gamma_{a}\gamma_{b}\gamma_{c}\gamma_{d}}{r_{ab}^{3}r_{cd}^{3}}\tau_{c}S^{2}\frac{1}{2}\left(3\cos^{2}\theta \left(ab,cd\right)-1\right),$$

(2) $$\Gamma_{ab,u}^{DP,CSA}\left(\theta \right)=\;\frac{4}{15}\frac{\mu_{0}\hbar }{4\pi }\frac{\gamma_{a}\gamma_{b}}{r_{ab}^{3}}B_{0}\gamma_{u}\tau_{c}S^{2}\frac{1}{2}\sum_{i=x,y,z}\sigma_{ii}^{u}(3\cos^{2}\theta \left(ab,u_{i})-1\right),$$

(3) $$\Gamma_{u,v}^{CSA,CSA}\left(\theta \right)=\;\frac{8}{45}B_{0}^{2}\gamma_{u}\gamma_{v}\tau_{c}S^{2}\frac{1}{2}\sum_{i,j=x,y,z}\sigma_{ii}^{u}\sigma_{jj}^{v}(3\cos^{2}\theta \left(u_{i},v_{j})-1\right).$$

**Figure 1:**
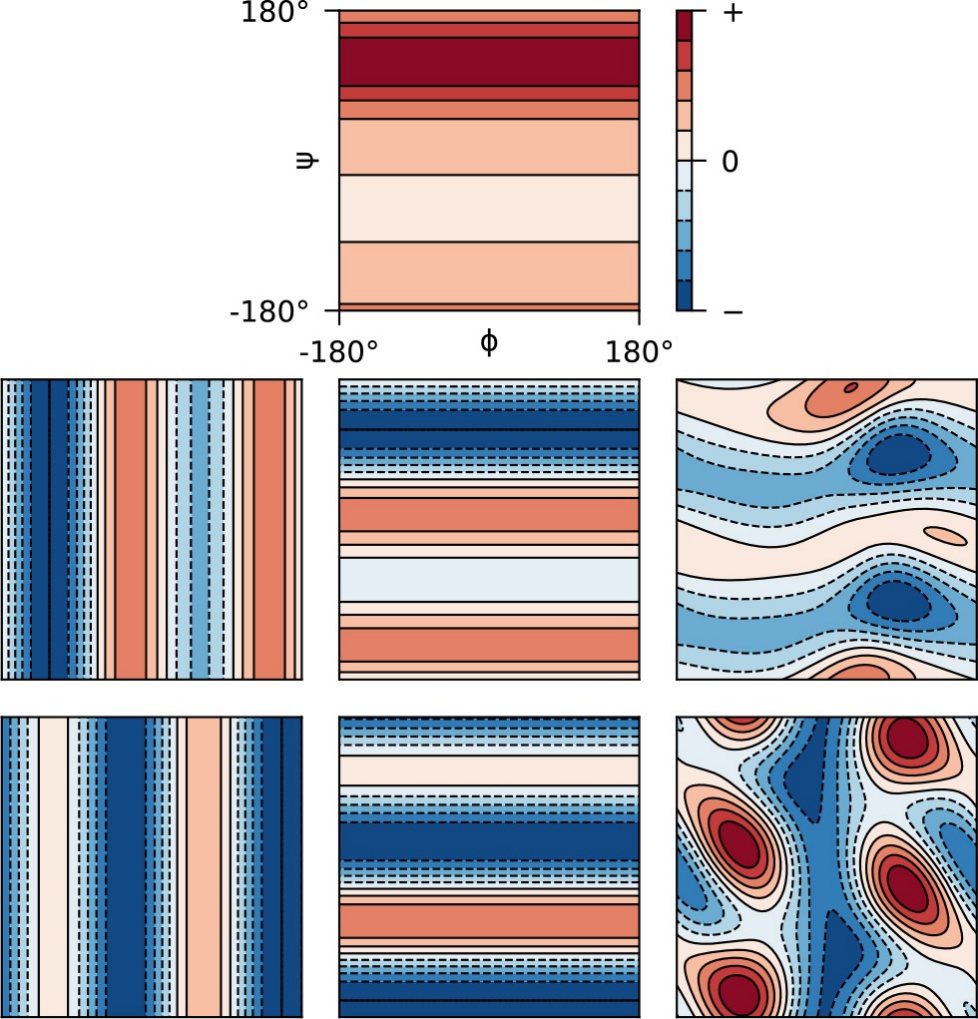
Angular dependencies of different dipole/CSA interferences assuming a rigid backbone geometry. Upper middle: $H_{i}^{\alpha }H_{i+1}^{N},C{{}^{'}}_{i}\left(\psi \right)$ . Middle left: $C_{i}^{\alpha }H_{i}^{\alpha },N_{i}H_{i}^{N}\left(\phi \right)$ . Middle: $C_{i}^{\alpha }H_{i}^{\alpha },N_{i+1}H_{i+1}^{N}\left(\psi \right)$ . Middle right: $H_{i}^{\alpha }H_{i}^{N},C{{}^{'}}_{i}\left(\phi ,\psi \right)$ . Lower left: $C_{i}^{\alpha }H_{i}^{\alpha },C{{}^{'}}_{i-1}\left(\phi \right)$ . Lower middle: $C_{i}^{\alpha }H_{i}^{\alpha },C{{}^{'}}_{i}\left(\psi \right)$ . Lower right: $N_{i}H_{i}^{N},C{{}^{'}}_{i}\left(\phi ,\psi \right)$ .

*a*, *b*, *c* and *d* denote nuclei subject to dipolar coupling, *u* and *v* are nuclei with CSA, *γ* is the gyromagentic ratio, *r* is the distance between two nuclei, *σ*_*xx*,*yy*,*zz*_ are the tensor components of the diagonal CSA tensor (in ppm), *μ*_0_ is the vacuum permeability, ℏ is the reduced Planck constant, *B*_0_ is the magnetic field strength, *τ_c_* is the overall correlation time, *S* ^2^ is the local order parameter and *θ* denotes the projection angle between the dipolar vectors (**ab**, **cd**) and/or the principal axes of the CSA tensor coordinate system of nucleus *u* ($u_{x}$ , $u_{y}$ , $u_{z}$ ) or nucleus *v* ($v_{x}$ , $v_{y}$ , $v_{z}$ ). These projection angles are of course related to the backbone geometry, allowing us to map $\Gamma \left(\theta \right)$ to Γ(ϕ,ψ) . Figure 1 shows the angular dependencies of all CCR rates employed in this work.

### 2.2 Maximum Entropy

We consider a distribution of backbone dihedral angles in (*φ*,*ψ*)‐space in terms of discretized populations $p_{\phi ,\psi }$ , with $\phi ,\psi \in [-180^{\circ },180^{\circ }[$ . The information about the $p_{\phi ,\psi }$ is represented by *m* population avagered CCR rates ${\tilde{\Gamma }}_{j}=\sum_{\phi ,\psi }p_{\phi ,\psi }\Gamma_{j}\left(\phi ,\psi \right)$ , j=1,...m . These observables might not be able to uniquely determine the underlying populations $p_{\phi ,\psi }$ . If this is the case, Jaynes’ Maximum Entropy (MaxEnt) principle^[38]^ identifies the optimal solution as the maximum entropy distribution compatible with the data. Following Jaynes’ original narrative, this principle can be derived from the intuitive notion of entropy as an information measure. Any other lower entropy solution would contain more information than originally supplied. While the original calculus has remained essentially unchanged, its justifications and axiomatizations have been thoroughly investigated over the years[^39^, ^40^, ^41^, ^42^, ^43^, ^44^, ^45^] and linked to the principles of Bayesian inference.[^46^, ^47^, ^48^]

Irrespective of its interpretation, the MaxEnt calculus takes the form of a constrained optimization problem, see e. g. Refs. [49–52] Jaynes’ originally suggested maximization of Shannon entropy^[53]^ is generalized in

(4) $$\begin{array}{c} \;\max_{p_{\phi ,\psi }}S\left(p_{\phi ,\psi }\right), \end{array}$$

where $p_{\phi ,\psi }$ is the probability vector and *S* is the negative Kullback‐Leibler divergence^[54]^ or relative entropy,

(5) $$\begin{array}{c} \;S\left(p_{\phi ,\psi }\right)=-\sum_{\phi ,\psi }p_{\phi ,\psi }\log\frac{p_{\phi ,\psi }}{q_{\phi ,\psi }}, \end{array}$$

where $q_{\phi ,\psi }$ denote the so‐called *prior* probabilities, which result from the relative nature of entropy measures in general. Analogous to its counterpart in Bayesian statistics, the prior is often interpreted as a state of *a priori* beliefs. Here, the prior probabilities $q_{\phi ,\psi }$ are not predefined but fixed, hence *S* is a function of the $p_{\phi ,\psi }$ alone.

In addition the $p_{\phi ,\psi }$ are subject to a set of constraints,

(6) $$1=\sum_{\phi ,\psi }p_{\phi ,\psi },$$

(7) $${\tilde{\Gamma }}_{j}=\sum_{\phi ,\psi }p_{\phi ,\psi }\Gamma_{j}\left(\phi ,\psi \right),\;j=1,...,m,$$

where (6) represents the normalization condition. As shown in the Appendix, the problem is solved by forming the Lagrangian (17) and inspecting its partial derivatives (18) with respect to $p_{\phi ,\psi }$ , which yields the general expression

(8) $$\begin{array}{c} \;p_{\phi ,\psi }\left(\lambda_{1},...,\lambda_{m}\right)=\frac{q_{\phi ,\psi }}{Z}\exp\left(\sum_{j=1}^{m}\lambda_{j}\Gamma_{j}\left(\phi ,\psi \right)\right), \end{array}$$

where Z is the *partition function*,

(9) $$\begin{array}{c} \;Z=\sum_{\phi ,\psi }q_{\phi ,\psi }\exp\left(\sum_{j=1}^{m}\lambda_{j}\Gamma_{j}\left(\phi ,\psi \right)\right), \end{array}$$

which results from the normalization condition (6). Note that the MaxEnt distribution (8) is a function solely of the *m* Lagrange multipliers *λ_j_*, hence the dimensionality of the original problem (4), (6), (7) is drastically reduced.

While the resulting system of *m* equations and *m* unknowns could readily be solved, a common strategy involves forming the so‐called *dual* Lagrangian instead.[^49^, ^55^, ^56^, ^57^] Substitution of (8) into the Lagrangian yields a convex “free energy” potential of $\lambda_{1},...,\lambda_{m}$ , see Apendix Eq. 23,

(10) $$\begin{array}{c} \;\min_{\lambda_{1},...,\lambda_{m}}L\left(\lambda_{1},...,\lambda_{m}\right)=\log\;Z\left(\lambda_{1},...,\lambda_{m}\right)-\sum_{j=1}^{m}\lambda_{j}{\tilde{\Gamma }}_{j}, \end{array}$$

which allows to solve the originally constrained problem (4), (6), (7) by unconstrained optimization of (10) instead. At its minimum, the partial derivatives of *L* equate to the conditions stated in 7,

(11) $$\begin{array}{c} \;0=\frac{\partial L}{\partial \lambda_{x}}=\frac{\partial \log Z}{\partial \lambda_{x}}-{\tilde{\Gamma }}_{x}=\left\langle \Gamma_{x}\right\rangle -{\tilde{\Gamma }}_{x}, \end{array}$$

where angled brackets denote the population weighted average over the MaxEnt distribution (8). The minimizing multipliers $\lambda_{1}\;{}^{*},...,\lambda_{m}\;{}^{*}$ uniquely characterize $p{{}^{*}}_{\phi ,\psi }\left(\lambda_{1}\;{}^{*},...,\lambda_{m}\;{}^{*}\right)$ (8) with maximum *S* (4) subject to the constraints (6) and (7). The Hessian of *L*,

(12) $$\begin{array}{c} \;\frac{\partial^{2}L}{\partial \lambda_{x}\partial \lambda_{y}}=\frac{\partial^{2}\log Z}{\partial \lambda_{x}\partial \lambda_{y}}=\left\langle \Gamma_{x}\Gamma_{y}\right\rangle -\left\langle \Gamma_{x}\right\rangle \left\langle \Gamma_{y}\right\rangle , \end{array}$$

corresponds to a positive semi‐definite covariance matrix^[49]^ or Fisher information metric.^[54]^ Minimization of (10) is thus straightforward to implement numerically.

### 2.3 Maximum Entropy Regression

At first glance, the MaxEnt framework appears like a natural fit for dealing with ensemble averaged data. Instead of modeling the populations $p_{\phi ,\psi }$ explicitly, they can be obtained from a simple and low‐dimensional optimization problem. However, we have not yet accounted for the possibility of errors in the data. This is a key limitation of MaxEnt in practice, because experimental information rarely takes the form of expectation constraints as assumed in (7). Instead, the conditional probability terms of Bayesian statistics are generally required to properly account for experimental uncertainties and/or inaccuracies of the forward model.^[58]^ However, defining and sampling meaningful priors and likelihoods over ensembles, i. e. distributions over distributions, is challenging both conceptually and practically. While this concept has been explored,[^59^, ^60^, ^61^] most pragmatic approaches opt for a simple point estimate instead.

Here, we suggest a novel perspective that aims to preserve the advantages of MaxEnt as introduced in Section 2.2, such as low dimensionality and well‐defined optimality conditions. To distinguish from noise‐ and error‐free constraints ${\tilde{\Gamma }}_{j}$ , we denote experimentally determined rates as ${\tilde{\Gamma }}_{j}^{\exp}$ . While the general notion of population averaging still applies, we acknowledge that the associated equality constraints (7) are ill‐suited for erroneous ${\tilde{\Gamma }}_{j}^{\exp}$ . Thus, a MaxEnt representation as in (8) cannot be derived from ${\tilde{\Gamma }}_{j}^{\exp}$ , at least in a strict sense. However, we suggest that the functional form of $p_{\phi ,\psi }\left(\lambda_{1},...,\lambda_{m}\right)$ (8) still holds some validity. Reconsidering the Dual Lagrangian (10), we note that the gradient (11) evaluates the difference between prediction and observation, i. e. the residual. Instead of requiring this term to be zero, we could choose to minimize a suitable norm, which allows us to reframe MaxEnt in terms of a simple regression model. For now, we consider a conventional $\chi^{2}$ fit,

(13) $$\begin{array}{c} \;\min_{\lambda_{1},...,\lambda_{m}}\chi^{2}\left(\lambda_{1},...,\lambda_{m}\right)=\frac{1}{2}\sum_{j=1}^{m}{\left(\frac{\left\langle \Gamma_{j}\right\rangle -{\tilde{\Gamma }}_{j}^{exp}}{\sigma_{j}}\right)}^{2}, \end{array}$$

where angled brackets denote the population weighted average over $p_{\phi ,\psi }\left(\lambda_{1},...,\lambda_{m}\right)$ (8) and *σ_j_* represents the standard deviation associated with Γ_*j*_. This expression is of course easily generalized to include multiple measurements of ${\tilde{\Gamma }}_{j}^{\exp}$ . The similarities with canonical MaxEnt are best illustrated by the optimality conditions,

(14) $$\begin{array}{c} \;0=\frac{\partial \chi^{2}}{\partial \lambda_{x}}=\sum_{j=1}^{m}\frac{1}{\sigma_{j}^{2}}\left(\left\langle \Gamma_{j}\right\rangle -{\tilde{\Gamma }}_{j}^{exp}\right)\frac{\partial \left\langle \Gamma_{j}\right\rangle }{\partial \lambda_{x}}, \end{array}$$

or equivalently, considering (11) and 12,

(15) $$\begin{array}{ccc} 0\; & =\; & \frac{\partial \chi^{2}}{\partial \lambda_{x}}=\sum_{j=1}^{m}\frac{1}{\sigma_{j}^{2}}\left(\frac{\partial \log Z}{\partial \lambda_{j}}-{\tilde{\Gamma }}_{j}^{exp}\right)\frac{\partial^{2}\log Z}{\partial \lambda_{x}\partial \lambda_{j}}\;\\ \; & =\; & \sum_{j=1}^{m}\frac{1}{\sigma_{j}^{2}}\left(\left\langle \Gamma_{j}\right\rangle -{\tilde{\Gamma }}_{j}^{exp}\right)\left(\left\langle \Gamma_{j}\Gamma_{x}\right\rangle -\left\langle \Gamma_{j}\right\rangle \left\langle \Gamma_{x}\right\rangle \right).\; \end{array}$$

The gradient entries (14) and (15) combine the gradient of the MaxEnt Dual (11) and its Hessian (12) in a sum of products, balancing the residuals of each rate weighted with their respective variances and covariances. Qualitatively speaking, if the residuals are approaching zero, i. e. a very good fit can be achieved, the *χ* ^2^ fit will resemble an orthodox MaxEnt solution. If this is not the case, the covariances are adjusted and scaled down in order to minimize the non‐zero residuals. Of course, the latter case bears little similarity with a conventional MaxEnt representation. Rather than increasing the inferential uncertainty, experimental errors lead to smaller covariances, implying averages over smaller subspaces and thus lower rather than higher entropy solutions. In a way, the expression for $p_{\phi ,\psi }\left(\lambda_{1},...,\lambda_{m}\right)$ (8) was used for its convenient parametrization without keeping its original justification intact. Thus, entropy has to be reintroduced to the equation, which can be achieved by regularization. Since explicit entropy has already been investigated by other studies,[^21^, ^23^, ^24^, ^62^, ^63^, ^64^] we choose to explore it implicitly using a more conventional quadratic regularization term commonly known in the context Tikhonov regularization[^65^, ^66^] or ridge regression,[^67^, ^68^]

(16) $$\begin{array}{c} \;\min_{\lambda_{1},...,\lambda_{m}}\chi_{reg}^{2}=\frac{1}{2}\sum_{j=1}^{m}{\left(\frac{\left\langle \Gamma_{j}\right\rangle -{\tilde{\Gamma }}_{j}^{exp}}{\sigma_{j}}\right)}^{2}+\frac{\beta^{2}}{2}\sum_{j=1}^{m}\lambda_{j}^{2}, \end{array}$$

where *β* is a free parameter, often identified as “temperature”, balancing the contributions of *χ* ^2^ and the L2 penalty. This indirect way of entropy penalization is particularly suited for *biasing* techniques[^22^, ^69^, ^70^, ^71^] that do not allow for explicit calculation of *S*. Its effect on the entropy can easily be assessed from Eq. (8), noticing that smaller *λ_j_* imply smaller perturbations of the prior. An established heuristic for the choice of *β* is the L‐curve criterion.[^72^, ^73^] As nonlinear least squares problems, both Eq. (13) and (16) are locally convex and straightforward to minimize using the Levenberg‐Marquardt algorithm.^[74]^

Naturally, Eq. (16) shares many similarities with previously devised approaches. Already in 1978, Gull and Daniell assumed reduced *χ* ^2^‐statistics to repurpose the MaxEnt Lagrangian for erroneous data.^[75]^ Other authors chose to preserve the Lagrangian by extending the equality constraints assuming additive errors, an idea first sketched in the 90 s[^76^, ^77^, ^78^] and recently rediscovered for structural biology purposes by Cesari et al.[^22^, ^70^] Assuming Gaussian errors, both approaches can be shown (Ref. [24] and [70], supplementary material) to correspond to the Bayesian MAP estimate of Hummer and Köfinger,[^21^, ^24^] who reframed their entropy‐regularized *χ* ^2^‐fitting procedure[^62^, ^63^, ^64^] in Bayesian terms assuming a Gaussian likelihood and an entropy‐inspired prior. A different perspective has been sketched by Dudik et al. who investigated the Lagrangian under various “relaxed” constraints to avoid overfitting.[^79^, ^80^] While the heuristic proposed here is derived from slightly different considerations, it does of course illustrate the same general principle. Uncertainty in the data must be reflected in a stronger emphasis on the prior.

## 3 Results

Eq. (16) depends on various parameters and assumptions which need to be carefully assessed before interpreting its solution, namely the variances $\sigma_{j}^{2}$ weighting different rates according to their overall size and spread, the regularization parameter *β* balancing experimental and prior information and the forward model relating the observed rates Γ_*j*_ to the backbone dihedral angles.

**Figure 2:**
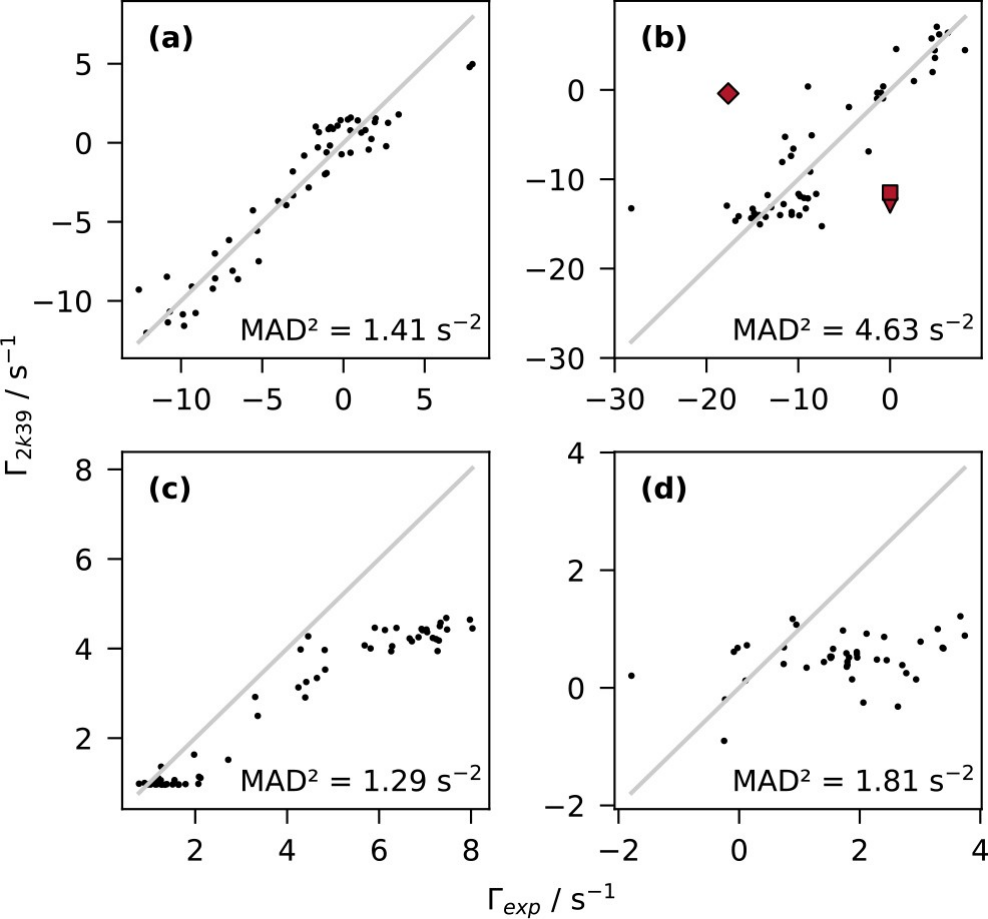
Comparison of CCR rates Γ_2*k*39_ calculated from the Lange ensemble,^[81]^ PDB code 2k39, and the experimentally obtained rates Γ_*exp*_, (a) 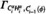, (b) $\Gamma_{C_{i}^{\alpha }H_{i}^{\alpha },N_{i+1}H_{i+1}^{N}}\left(\psi \right)$ , (c) $\Gamma_{H_{i}^{\alpha }H_{i+1}^{N},C_{i}^{'}}\left(\psi \right)$ , (d) $\Gamma_{H_{i}^{\alpha }H_{i}^{N},C_{i}^{'}}\left(\phi ,\psi \right)$ . The squared median absolute deviations *MAD* ^*2*^ are specified in the lower right corners. Outliers of rate (b) that were found critical in the subsequent fitting procedure are highlighted in red. Diamond: 30I, Triangle: 41Q, Square: 70V. The remaining three rates are depicted in Figure S1, Supporting Information.

In a first step, the variances $\sigma_{j}^{2}$ of each rate were assessed by comparing the experimental CCR rates with rates predicted from the Ubiquitin ensemble of Lange et al.,^[81]^ PDB code 2k39. Simulating the general case of limited prior knowledge, CSA tensors and dynamics were assumed uniform for all rates and residues. With a global correlation time *τ_c_* of 4.1 ns, an overall order parameter *S* ^2^ of 0.7 (similar to Refs. [19, 82]) was found to give reasonable but far from perfect agreements between measured and predicted rates. Four examples are depicted in Figure 2, the remaining rates are shown in Figure S1, Supporting Information. Accounting for differences in temperature and/or magentic field strength, the obtained range of experimental values agrees well with the original publications validated on Ubiquitin.[^14^, ^28^, ^29^, ^30^, ^32^] As can be seen from Figure 2, not all measured rates can safely be modeled assuming simple Gaussian noise. Since the conventional variance estimate is highly sensitive to outliers, the squared median absolute deviation (MAD^2^) was considered a better suited estimate for the subsequent fitting procedure. Due to the noticable presence of outliers, systematic deviations and the poor agreements of rate (d), $H_{i}^{\alpha }\;H_{i}^{N}/\;C_{i}^{'}\left(\phi ,\psi \right)$ , the reported minimum requirement of five observables per residue^[13]^ was applied. For 56 out of 74 non‐terminal residues five or more CCR rates could be quantified. Residues that yielded four or less CCR rates (18, including 5 glycines and 3 prolines) were excluded from the analysis.

**Figure 3:**
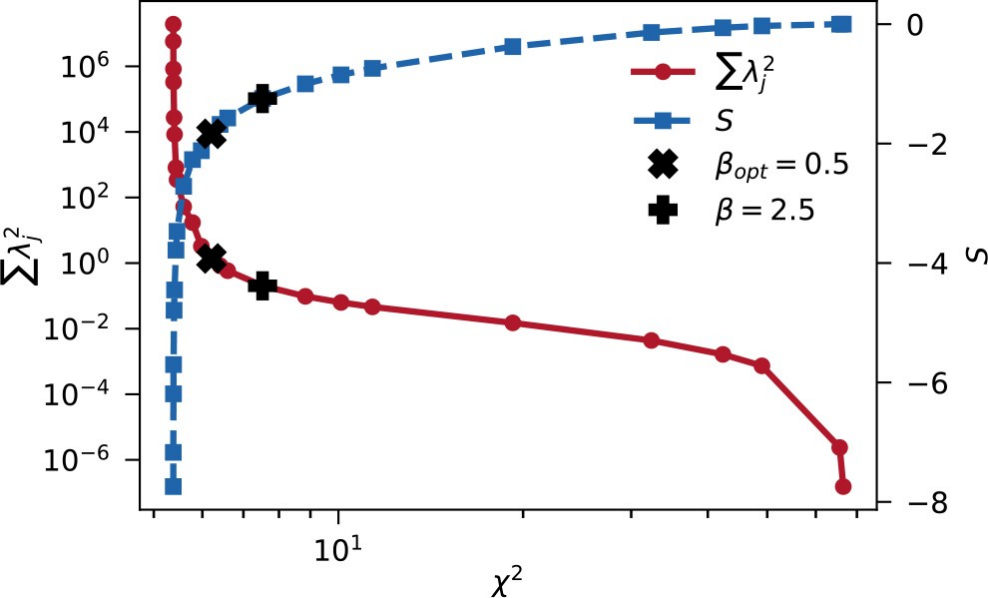
Red circles: Exemplary L‐curve for I14 obtained by optimizing $\chi_{reg}^{2}$ , Eq. (16), for different *β* (between 0 and 1000) and $S^{2}=0.7$ . The contributions of *χ* ^2^ and $\sum \lambda_{j}^{2}$ describe an L‐shaped curve in a log‐log plot. Blue squares: The entropy *S*, Eq. (5), of the backbone dihedral angle distribution, Eq. (8), corresponding to the minimum of $\chi_{reg}^{2}$ . Points are linearly interpolated for improved readability. The knee‐point at $\beta_{opt}=0.5$ is highlighted, β=2.5 marks the regularization parameter chosen for further evaluations, see Figure 4, 5 and 6.

With *τ_c_*, *S* ^2^ and $\sigma_{j}^{2}$ fixed, the regularization parameter *β* is the only free parameter left. It is often chosen using the L‐curve criterion. For each residue, $\chi_{reg}^{2}$ is minimized for different *β* using the random coil prior defined in Sec. 7. The pairwise contributions of *χ* ^2^ and $\sum \lambda_{j}^{2}$ are plotted on a logarithmic scale. The knee point of the curve indicates the region where *χ* ^2^ and $\sum \lambda_{j}^{2}$ are balanced in the sense that lower *β* allow for high *λ*‐variability with little gain in *χ* ^2^ while higher *β* restrain the *λ_j_* at noticeable expense of *χ* ^2^. An exemplary L‐curve for I14 is shown in Figure 3.

**Figure 4:**
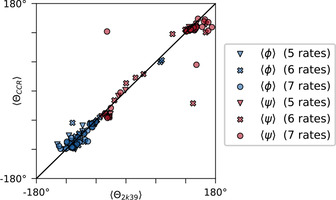
Comparison of average backbone dihedral angles $\left\langle \theta \right\rangle$ in Ubiquitin between the Lange ensemble,^[81]^ PDB code 2k39, and the CCR‐derived (ϕ,ψ )‐distributions obtained from Eq. (16) with $S^{2}=0.7$ and β=2.5 . As specified in the legend, different markers are used to indicate the number of experimental CCR rates used. The three strong *ψ*‐outliers correspond to the outliers of $\Gamma_{C_{i}^{a}H_{i}^{a},N_{i+1}H_{i+1}^{N}}\left(\psi \right)$ highlighted in Figure 2, panel (b).

However, considering the quality of the data and the obvious presence of outliers, Figure 2, the L‐curve criterion is likely too optimistic in favoring low *χ* ^2^ solutions. This was confirmed by comparing the fitting results with the Lange ensemble^[81]^ in terms of average dihedral angles for different choices of *β*, Figure S2, Supporting Information, bottom row. By evaluating the L‐curves collectively, a more conservative estimate was found in the highest overall knee point of β=2.5 . The corresponding fitting results are summarized in Figure 4.

As entropy *S* and $\sum \lambda_{j}^{2}$ are defined relative to a prior distribution, it is important to assess the influence of the random coil prior used. Thus, the fitting procedure was repeated using the uniform prior. The results are summarized in Figure S3, Supporting Information. Compared to the random coil prior, Figure S2, Supporting Information, the uniform prior is less capable of correcting for improbable (*φ*,*ψ*)‐assignments.

**Figure 5:**
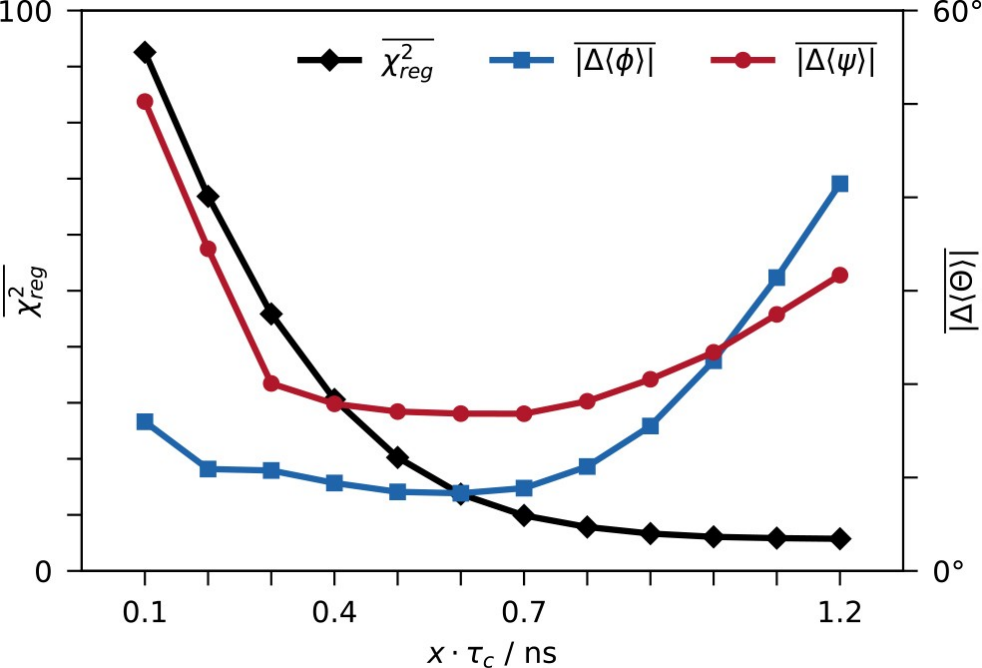
Average fitting results of Eq. (16) with *β*=2.5 for all 56 residues with five or more quantifiable CCR rates assuming different dynamic scalings *x* ≙ *S* ^2^ and *τ_c_*=4.1 ns. $\left|\Delta \left\langle \theta \right\rangle \right|$ is calculated with respect to the average backbone dihedral angles of the Lange ensemble,^[81]^ PDB code 2k39. Points are linearly interpolated for improved readability.

Finally, the forward model itself represents an additional source of uncertainty. The functional forms of Eq. (1) and (2) build on the assumption of isotropic molecular tumbling in the absence of internal dynamics, which leads to the convenient factorization of structural and dynamical contributions. To asses the influence of the assumed dynamic scaling, the order parameter *x* ≙ *S* ^2^ was varied between 0.1 and 1.2, as shown for β=2.5 in Figure 5. A comparison of fitting results with different *x* and *β* is shown in Figure S2, Supporting Information.

**Figure 6:**
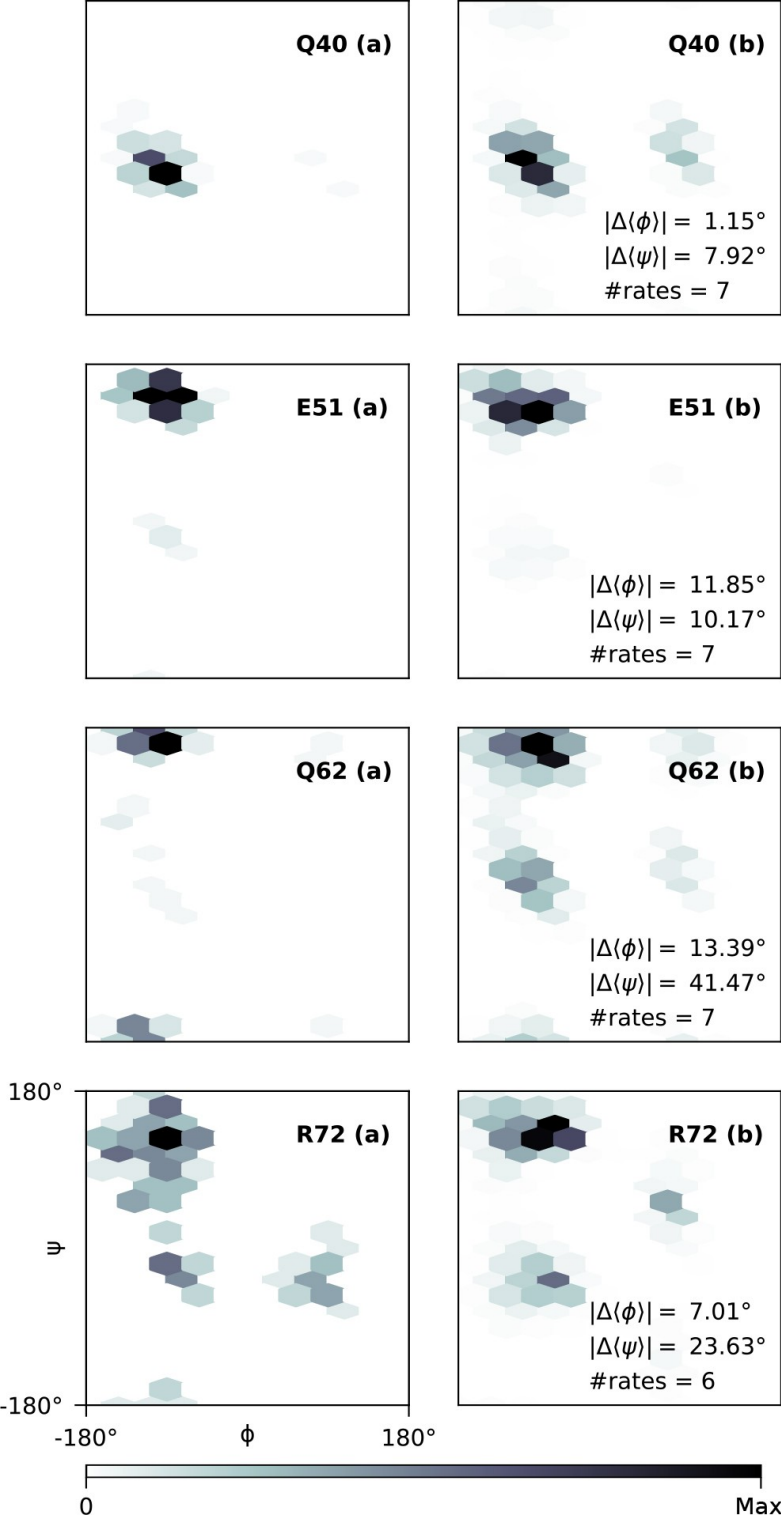
Comparison of selected backbone dihedral angle distributions in Ubiquitin between the Lange ensemble^[81]^ in column (a) and the CCR‐derived fitting results in column (b) obtained from Eq. (16) with $S^{2}=0.7$ and β=2.5 . Populations are color‐coded according to the linear color gradient at the bottom. Residue type and number are indicated in the top right corner. $\left|\Delta \left\langle \phi \right\rangle \right|$ and $\left|\Delta \left\langle \psi \right\rangle \right|$ denote the absolute difference in average dihedral angles between (a) and (b), #rates is the number of CCR rates used to derive (b).

To evaluate *φ* and *ψ* not only in terms of averages, a selection of flexible residues is compared to the ensemble of Lange et al.^[81]^ in terms of (*φ*,*ψ*)‐distributions in Figure 6. The full set of (*φ*,*ψ*)‐distributions is shown in Figure S4, Supporting Information.

## 4 Discussion

In Figure 2, a selection of experimentally determined rates is compared to their expected values calculated from the ensemble of Lange et al.^[81]^ While overall correlation between experiments and predictions can be observed, there are obvious differences between different rates. For (a), $C_{i}^{\alpha }H_{i}^{\alpha }$ /$C_{i-1}^{'}\left(\phi \right)$ , deviations are relatively small and could be reasonably approximated by Gaussian noise. The spread of (b), $C_{i}^{\alpha }H_{i}^{\alpha }$ /$N_{i+1}H_{i+1}^{N}\left(\psi \right)$ , is not only larger but appears to contain a few obvious outliers, three of which have been found to heavily influence the subsequent analysis (highlighted in red). Our newly proposed experiment^[14]^ (c), $H_{i}^{\alpha }H_{i+1}^{N}$ /$C_{i}^{'}\left(\psi \right)$ , shows systematic deviations from predicted values most pronounced for higher rates. This is consistent with our previous findings, suggesting a higher overall correlation time as well as a strong sensitivity to variations of the CSA tensor and/or the backbone geometry.^[14]^ Rate (d), $H_{i}^{\alpha }H_{i}^{N}$ /$C_{i}^{'}\left(\phi ,\psi \right)$ , shows particularly poor agreements. The expected angular dependency is obscured by considerable scatter and systematic deviations, likely due to its small functional range with the highest sensitivity at mostly unpopulated regions with positive *φ*, see Figure 1. The three remaining rates (e), (f) and (g), Figure S1, Supporting Information, behave similarly to (a) and (c). Errors appear mostly randomly distributed, minor systematic biases can be observed for rates (e), $N_{i}H_{i}^{N}$ /$C_{i}^{'}\left(\phi ,\psi \right)$ , and (g), $C_{i}^{\alpha }H_{i}^{\alpha }$ /$C_{i}^{'}\left(\psi \right)$ .

Considering these quantitative discrepancies, the agreements in terms of MaxEnt‐derived $\left\langle \phi \right\rangle$ and $\left\langle \psi \right\rangle$ , Figure 4, might appear quite surprising. However, as established in Sec. 3.3, the experimental and theoretical uncertainties are reflected in a stronger emphasis on the prior (β=2.5 ). Instead of forcing *χ* ^2^ close to its absolute minimum, a suitable balance between *χ* ^2^ and $\sum \lambda_{j}^{2}$ allows to suppress overfitting, revealing the complementary structural dependencies of CCR rates even in the presence of systematic and experimental errors.

To achieve this balance, i. e. finding an appropriate *β*, the L‐curve offers an intuitive heuristic. As explained in Sec. 4, the pairwise contributions of *χ* ^2^ and $\sum \lambda_{j}^{2}$ for different *β* are compared on a log‐log plot as illustrated for I14 in Figure 3. At the knee point, *λ*‐variability is considered sufficiently penalized without excessive restraint of *χ* ^2^. However, these knee point solutions, with *β* between 0.01 and 2.5 for different residues, do not necessarily yield the best results, see Figure S2, Supporting Information, bottom row. Instead, we find that a more strongly weighted prior of β=2.5 for all residues leads to better agreements in terms of average dihedral angles. As described in Sec. 4, the presence of errors should be reflected by an increase in inferential uncertainty, yielding higher rather than lower entropy solutions. Thus, a low *χ* ^2^ does not necessarily indicate a good solution, especially if it is achieved at the expense of an informative prior. While the L‐curve provides a useful visual guideline for choosing *β*, the knee point criterion appears too reliant on *χ* ^2^ to avoid overfitting, prior information needs to be considered more explicitly. Alternatively, the observed change in slope of *S* or $\sum \lambda_{j}^{2}$ , i. e. the second derivative, might provide a reasonable heuristic.

Of course, this does not imply that the prior can substitute for the experimental information encoded in *χ* ^2^, the experimental data still determines the solution. This is well illustrated by the highlighted outliers of rate (b), $C_{i}^{\alpha }H_{i}^{\alpha }$ /$N_{i+1}H_{i+1}^{N}\left(\psi \right)$ , Figure 2. As can be seen by their vertical and horizontal projections, these points correspond to very plausible values for *ψ*. As a consequence, these discrepancies are mirrored by the obvious $\left\langle \psi \right\rangle$ ‐deviations in Figure 4. Upon exclusion of rate (b), $C_{i}^{\alpha }H_{i}^{\alpha }$ /$N_{i+1}H_{i+1}^{N}\left(\psi \right)$ , the predicted (*φ*,*ψ*)‐densities improve drastically (Figure S5, Supporting Information). The prior can only correct for implausible conformations, *χ* ^2^ still shapes the solution. What conformations are deemed implausible depends on the prior of course. See Figure S2 and S3, Supporting Information, to compare the fitting results between random coil and uniform prior.

These findings suggest another interesting implication. If the absolute value of *χ* ^2^ is of minor importance, how crucial is the choice of the effective correlation time? This question is explored in Figure 5, which summarizes the average fitting results assuming “order parameters” *x* ≙ *S* ^2^ between 0.1 and 1.2. Here, the “order parameter” simply serves to rescale the functional range of Γ. Thus, it is not surprising that the average $\chi_{reg}^{2}$ increases with lower *x*. However, average $\left\langle \phi \right\rangle$ and $\left\langle \psi \right\rangle$ still appear quite stable over a considerable range, illustrating the general robustness with respect to systematic errors. As long as the angular dependencies (1), (2) and (3) are reasonably applicable, their overall scale is of less importance. However, if the scaling is underestimated by too much (*x*=0.1–0.3), the data might point towards a different (*φ*,*ψ*)‐region, analogous to the outliers in Figure 4. In Figure S2, Supporting Information, this increase of incorrectly assigned (*φ*,*ψ*)‐pairs can readily be seen. Overestimating the dynamic scaling (*x*=0.9–1.2) leads to an entirely different behavior. As can be seen by the vertical spreads in Figure S1 and S2, Supporting Information, increasing the functional range of Γ puts less restraint on (*φ*,*ψ*)‐space, allowing for better fits in terms of $\chi_{reg}^{2}$ . However, the underlying (*φ*,*ψ*)‐distributions can be implausibly distorted as a consequence.

These results suggest that the dynamical contributions, attributed to a simple effective correlation time, are less crucial than the angular information encoded in locally concerted motions. Of course, dynamics and geometry are more intertwined in reality. For IDPs in particular, more pronounced diffusion anisotropy and local dynamics will lead to even further deviations from the simple angular dependencies assumed in Eqs. (1), (2) and (3). Still, a surprising robustness can be observed for Ubiquitin despite its notable deviations from theory, Figure 2. While the assumed dynamic model must be verified and possibly adapted for other protein systems, this robust fitting behavior might hold true in the general case. To this end, we expect that Figure 5 could be used similarly to the L–Curve, Figure 3, by evaluating the relative changes in $p\left(\phi ,\psi \right)$ and $\chi_{reg}^{2}$ upon variation of the dynamics.

Finally, to compare *φ* and *ψ* in terms of actual distributions, a selection of flexible residues is compared to the Lange ensemble^[81]^ in Figure 6.

Q40 is part of a turn‐motif preceding a *β*‐annotated segment from 41 to 45. While featuring mostly right‐handed helical propensities in the PDB ensemble (a), there is noticeable density in the left‐handed region as well. This feature is reproduced by the CCR‐derived density (b), albeit with slightly different populations. Still, $\left\langle \phi \right\rangle$ and $\left\langle \psi \right\rangle$ are in very good agreement. In addition, the CCR data seems to suggest a slight *β*‐propensity, which might be noteworthy considering the transition from turn to *β*‐strand.

E51 has no assigned secondary structure motif, but precedes a short turn segment, indicated by a small *α*‐propensity in the PDB ensemble (a) alongside a broadly populated *β*‐region. Again, the CCR‐derived densities are in strong qualitative and quantitative agreement.

Q62 is similar to E51. It has no assigned secondary structure but is located right before a short turn segment. However, in terms of $\left\langle \phi \right\rangle$ and $\left\langle \psi \right\rangle$ , the agreement between the PDB ensemble (a) and the CCR‐derived populations (b) appears rather poor. Still, the CCR fit (b) is quite remarkable. Broad *β*‐populations extending to negative *ψ* are well reproduced. Propensities of *α* and *ζ* ^[83]^ ($\phi \approx -120{}^{\circ }$ , $\psi \approx 70{}^{\circ }$ ) in (a) are correctly predicted but more heavily weighted in (b). Interestingly, even the small *ϵ*‐populations around $\phi =50{}^{\circ }$ and $\psi =-180{}^{\circ }$ are reproduced, which is expected to be very sparsely populated for non‐glycine residues. This presence of positive *φ* likely explains the noticeable and incorrect emphasis on left‐handed *α* in (b), which is far more frequent in the prior than *ϵ* and thus more pronounced.

R72 is annotated as bend‐like and part of the flexible C‐terminal tail. It follows a *β*‐strand stretch from 66–71. Its flexible nature is well‐reflected in the PDB ensemble (a) with broadly distributed populations in *β*, *ζ* and *α*‐regions. Interestingly, propensities with positive *φ* are not centered around canonical left‐handed *α*, but rather shifted towards smaller values of *ψ*. Overall, these propensities are well reproduced by the CCR‐derived distribution (b) with similar densities of *β* and right‐handed *α*. However, (b) appears far smoother than (a), a result of comparing 116 snapshots to a comparatively fine‐grained distribution. In addition, little *ζ*‐propensity is found in (b) and positive *ψ* values are biased towards the left‐handed *α*‐region, again illustrating the influence of the chosen prior.

As expected, average *φ* and *ψ* are not perfectly representative for (*φ*, *ψ*)‐distributions especially in flexible residues. While noticeable deviations might be observed on average, there are still similarities to be discovered in detail. Overall, CCR‐guided MaxEnt reweighting appears well suited to characterize rigid and flexible residues alike. However, details can only be resolved as far as experiments and prior allow. Occasional artifacts of experimental deviations and conflicting prior assumptions are still noticeable and should be addressed with care. Conformational heterogeneity can reflect structural flexibility as well as inferential uncertainty, i. e. rigid residues can appear rather flexible, see Figure S4, Supporting Information. Including additional observables, such as CCR rates, scalar couplings, chemical shifts or short‐range NOEs, can be expected to alleviate these effects. Quantitative agreements could be further improved by multiple measurements and/or alternative pulse sequences to allow for outlier corrections, cross‐validation and improved variance estimation.

In more general terms, it is important to emphasize that the information contained in CCR rates strongly depends on the prior employed. Detailed structural priors allow us to refine, calculate and/or analyze structural ensembles of folded proteins in terms of their dynamics.[^17^, ^18^, ^19^, ^20^] Accurate models of both structure and dynamics make the encoded CSA tensors accessible.[^15^, ^16^] The MaxEnt approach presented here provides a framework for the interpretation of CCR rates in cases of unspecific prior knowledge. While structural interpretations appear limited to backbone dihedral angle distributions as a consequence, we foresee a variety of possible extensions. Firstly, the interresidual CCR rate $C_{i}^{\alpha }H_{i}^{\alpha }$ /$C_{i+1}^{\alpha }H_{i+1}^{\alpha }\left(\psi_{i},\phi_{i+1}\right)$ might allow for joint analysis of sequential residues that would otherwise be treated independently. Secondly, by translating ensemble averaged observables into distributions over so‐called “collective variables”, our method might provide valuable inputs for metadynamics‐based MaxEnt biasing techniques.[^71^, ^84^, ^85^] Thirdly, our approach could of course be adapted to reweight a molecular dynamics simulation.^[25]^ For this case in particular we expect CCR rates to provide valuable experimental constraints reflecting both structure and dynamics.

## 5 Conclusions

Cross‐correlated relaxation (CCR) rates were shown to resolve dihedral angle distributions of both rigid and flexible residues in Ubiquitin. This was achieved using a modern Maximum Entropy (MaxEnt) reweighting approach that allows to account for the presence of structural flexibility in proteins.

While classical MaxEnt is ill‐equipped to deal with the errors that come with experimental data, we show that it can be recast into a simple regression scheme, retaining its low dimensionality and well‐defined optimality conditions. Crucially, the procedure does not depend on the number of ensemble members but only on the number of Lagrange multipliers, one for each experiment. This implicit way of modeling both entropy and conformational space proves to be very robust even outside its canonical scope. While the classical Lagrangian is not strictly applicable, it can still be repurposed to approximate the Lagrange multipliers. While many approaches achieve this by loosening the equality constraints, we derived a simple and robust *χ* ^2^‐type cost function which we expect to be particularly useful for MaxEnt *biasing* techniques that still build on rather cautious modifications of the Lagrange Dual.[^22^, ^69^, ^70^, ^71^]

However, since MaxEnt requires errors to be treated somewhat ad hoc to arrive at a simple point estimate, free parameters must always be treated with care. Firstly, the accuracy of each experiment is represented by a variance parameter to weight the contribution of each observable. While easy to estimate post hoc for a system like Ubiquitin, studies of less well‐known proteins might require additional considerations. Secondly, the balance between residual and prior must be assessed with care. We have found that the widely‐used L‐curve criterion might be too biased towards small residuals. Our data suggests that entropy or suitable estimates thereof are better suited to properly avoid overfitting. This aspect is particularly important if the fitting results cannot be compared with independent data. Thirdly, the prior itself must not be overlooked. Since overfitting is suppressed by enforcing entropy, the prior conformations necessarily affect the solution. While a uniform prior merely flattens the predicted densities, more informed priors put stronger emphasis on regions deemed probable a priori. For Ubiquitin, this does not mean that a random coil prior necessarily leads to broad distributions for rigid residues. However, an artificial bias towards more strongly populated regions in (*φ*,*ψ*)‐space could be observed for residues with unusual propensities especially with positive *φ*. Assessing the influence of different priors is thus highly recommended.

Lastly, the forward model relating experimental data to structural information is of crucial importance. In this work, we examined Ubiquitin under the oversimplified assumption of isotropic molecular tumbling without internal dynamics. Most notably, this implies that dynamical and structural contributions factorize, reducing the effect of dynamics to a simple scaling factor. While this model did not yield quantitative agreements of comparable quality between different CCR rates, the underlying (*φ*,*ψ*)‐distributions could still be resolved with surprising levels of detail, despite the presence of outliers and systematic deviations. These results suggest that the structural information encoded in CCR rates is capable of outweighing other sources of uncertainty, such as experimental errors, CSA tensor variations, simplified backbone geometries and imprecise dynamics. This robustness was observed even for residues with considerable amounts of conformational flexibility, indicating the unharnessed potential of CCR rates for studying disordered protein systems. While the simplified model of a rigid protein under isotropic tumbling must still be critically assessed and possibly adapted, we expect that CCR rates will allow us to better understand and potentially disentangle the subtle interplay of structure and dynamics in intrinsically disordered proteins.

## 6 Computational Methods

A total of seven different CCR interactions were employed, including our newly proposed experiment probing $\Gamma_{H_{i}^{\alpha }\;H_{i+1}^{N},\;\;C_{i}^{'}}\left(\psi \right)$ ^[14]^ and the closely related $\Gamma_{H_{i}^{\alpha }\;H_{i}^{N}\;,C_{i}^{'}}\left(\phi ,\psi \right)$ interference. The remaining five interactions have been described elsewhere: $\Gamma_{C_{i}^{\alpha }\;H_{i}^{\alpha }\;,N_{i+1}\;H_{i+1}^{N}}\left(\psi \right)$ ,^[27]^ $\Gamma_{C_{i}^{\alpha }\;H_{i}^{\alpha }\;,C_{i}^{'}}\left(\psi \right)$ ,^[28]^ $\Gamma_{C_{i}^{\alpha }\;H_{i}^{\alpha }\;,N_{i}\;H_{i}^{N}}\left(\phi \right)$ ,^[29]^ $\Gamma_{C_{i}^{\alpha }H_{i}^{\alpha },C_{i-1}^{'}}\left(\phi \right)$ ,^[30]^ $\Gamma_{N_{j}\;H_{i}^{N}\;,C_{i}^{'}}\left(\phi ,\psi \right)$ .^[32]^ To ensure internal consistency and reproducibility, all $\Gamma_{j}\left(\phi ,\psi \right)$ were calculated according to Eq. (1) and (2) by rotating an Avogadro^[86]^‐generated backbone geometry with $\phi ,\psi ,\omega =-180{}^{\circ }$ (Table S1, Supporting Information). Parameters were adapted primarily from Engh and Huber,^[87]^ angles involving hydrogens were taken from Momany et al.^[88]^ The principal axes of the carbonyl CSA tensor were set according to Teng et al.^[89]^ The Z‐axis was defined as the cross product of the $C^{'}$ ‐*O* and the $C^{'}$ ‐$C^{\alpha }$ bond unit vectors, the X‐ and Y‐axis as clockwise rotations of the $C^{'}$ ‐*O* bond unit vector around the Z‐axis by 82° and −8°, effectively approximating the *O*‐$C^{'}$ ‐*N* angle with 120°. The tensor components of Ubiquitin were taken from Cisnetti et al.^[15]^ *σ_xx_* and *σ_zz_* were set according to the reported averages as 249.4 ppm and 87.9 ppm. Following the suggested calibration, the average *σ_yy_* was obtained from the chemical shifts (BMRB ID 17769^[90]^) as 191.1 ppm. The resulting angular dependencies are depicted in Figure 1. A correlation time *τ_c_* of 4.1 ns was assumed,^[91]^ *S* ^2^ was treated as a free parameter. *B*_0_ was set in accordance with the experimental magnetic field of 600 MHz.

For quantitative comparison the ensemble of Lange et al.^[81]^ was used (PDB code 2k39). CCR rates were calculated from *φ* and *ψ* for every structure according to Eq. (1) and (2) using the above stated CSA tensor, backbone geometry and *τ_c_* assuming an order parameter *S* ^2^ of 0.7. Subsequent averaging over all 116 structures yielded the CCR rate predictions.

For MaxEnt regression according to Eq. (16), the variances $\sigma_{j}^{2}$ were estimated by the median absolute deviation between the 2k39‐based predictions and the experimental values. The CCR rates were approximated as 360x360 arrays by rotating the backbone (Table S1, Supporting Information) in 1° steps. Analogously, the random coil prior was defined on a 360x360 grid using the coil library of Manstyzov et al.^[92]^ After discarding ill‐defined terminal segments as well as glycine and proline residues, a total of 147091 angle pairs was extracted and rounded to integers. The prior was then obtained as the normalized histogram of angle pair counts. It is shown in Figure S6, Supporting Information. Out of 76 residues, 56 yielded five or more CCR rates. These residues were analyzed according to Eq. (16) with different *β* (0 to 1000) and *S* ^2^ (0.1 to 1.2). Minimization was achieved using the Levenberg‐Marquardt algorithm implemented in SciPy^[93]^ version 1.0.0 with iteratively updated scaling.^[94]^

## Experimental Section

The sample of 2 mM ${}^{13}$ C,${}^{15}$ N‐uniformly labeled Ubiquitin dissolved in 50 mM phosphate buffer of pH=7.0 , was purchased from Giotto Biotech.

All experiments were performed on a Bruker 600 MHz spectrometer equipped with a 5 mm room‐temperature probe. The experiments were performed at 298 K. The experimental parameters are summarized in Table 1.

**Table 1:** Experimental parameters for all data sets (dim ‐ dimensionality of the experiment, sw ‐ spectral width, td ‐ number of points acquired in a given dimension (real plus imaginary), version: J‐res ‐ J‐resolved type of experiment, ref/cross ‐ reference/transfer version of quantitative gamma experiment type, ns ‐ number of scans).

CCR rate	T_*c*_ [ms]	dim	C’		N		version	ns	time
			sw [Hz]	td	sw [Hz]	td			
$\Gamma_{C_{i}^{\alpha }H_{i}^{\alpha },C_{i-1}^{'}}\left(\phi \right)$	28	3D	2600	90	2300	140	J‐res	8	37 h
$\Gamma_{C_{i}^{\alpha }H_{i}^{\alpha },C_{i}^{'}}\left(\psi \right)$	28	3D	2600	96	2300	104	J‐res	4	14 h
$\Gamma_{C_{i}^{\alpha }H_{i}^{\alpha },N_{i+1}H_{i+1}^{N}}\left(\psi \right)$	28	2D	–	–	2127	96	ref	32	1 h
							cross	256	8 h
$\Gamma_{C_{i}^{\alpha }H_{i}^{\alpha },N_{i}H_{i}^{N}}\left(\phi \right)$	28	2D	–	‐	2127	220	ref	16	1 h
							cross	128	8 h
$\Gamma_{H_{i}^{\alpha }H_{i+1}^{N},C_{i}^{'}}\left(\psi \right)$	28	2D	–	–	2300	670	ref	4	1 h
							cross	48	12 h
$\Gamma_{H_{i}^{\alpha }H_{i}^{N},C_{i}^{'}}\left(\phi ,\psi \right)$	28	2D	–	–	1850	120	ref	48	2 h
							cross	384	16 h
$\Gamma_{N_{i}H_{i}^{N},C_{i}^{'}}\left(\phi ,\psi \right)$	66	3D	1850	238	2200	192	J‐res	4	67 h

The pulse sequence programs were prepared for Bruker spectrometers, according to the original publications: $\Gamma_{H_{i}^{\alpha }\;H_{i+1}^{N},\;C_{i}^{'}}\left(\psi \right)$ ,^[14]^ $\Gamma_{C_{i}^{\alpha }\;H_{i}^{\alpha },\;N_{i+1}\;H_{i+1}^{N}}\left(\psi \right)$ ,^[27]^ $\Gamma_{C_{i}^{\alpha }\;H_{i}^{\alpha },\;\;C_{i}^{'}}\left(\psi \right)$ ,^[28]^ 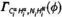,^[29]^ $\Gamma_{C_{i}^{\alpha }\;H_{i}^{\alpha },\;\;C_{i-1}^{'}}\left(\phi \right)$ ,^[30]^ $\Gamma_{N_{i}\;H_{i}^{N},\;\;C_{i}^{'}}\left(\phi ,\psi \right)$ .^[32]^ For some pulse sequences minor optimizations were introduced. These will be described in a future publication alongside the pulse sequence used to measure $\Gamma_{H_{i}^{\alpha }\;H_{i}^{N},\;\;C_{i}^{'}}\left(\phi ,\psi \right)$ . All pulse sequences are available from the authors upon request.

All experiments were performed using the conventional sampling scheme. Data were processed using the fast Fourier transform algorithm implemented in mddnmr.^[95]^ The data were displayed and analyzed using Sparky.^[96]^

## 7 Appendix

The constrained optimization problem (4),(6),(7) is recast into the Lagrangian

(17) $$\begin{array}{c} \;ℒ=-\sum_{\phi ,\psi }p_{\phi ,\psi }\log\frac{p_{\phi ,\psi }}{q_{\phi ,\psi }}+\sum_{j=1}^{m}\lambda_{j}\left(\sum_{\phi ,\psi }p_{\phi ,\psi }\Gamma_{j}\left(\phi ,\psi \right)-{\tilde{\Gamma }}_{j}\right) \end{array}+\lambda_{0}\left(\sum_{\phi ,\psi }p_{\phi ,\psi }-1\right).$$

The partial derivative with respect to $p_{\phi ,\psi }$ is

(18) $$\begin{array}{c} \;\frac{\partial ℒ}{\partial p_{\phi ,\psi }}=-\log\;p_{\phi ,\psi }-1+\log\;q_{\phi ,\psi }+\sum_{j=1}^{m}\lambda_{j}\Gamma_{j}\left(\phi ,\psi \right)+\lambda_{0}. \end{array}$$

Setting the partial derivative to zero, we obtain

(19) $$\begin{array}{c} \;\log\;p_{\phi ,\psi }=\log\;q_{\phi ,\psi }-1+\lambda_{0}+\sum_{j=1}^{m}\lambda_{j}\Gamma_{j}\left(\phi ,\psi \right). \end{array}$$

Taking the exponential and rearranging yields

(20) $$\begin{array}{c} \;p_{\phi ,\psi }=\frac{q_{\phi ,\psi }}{\exp\left(1-\lambda_{0}\right)}\exp\left(\sum_{j=1}^{m}\lambda_{j}\Gamma_{j}\left(\phi ,\psi \right)\right). \end{array}$$

By applying the normalization condition 6,

(21) $$\begin{array}{c} \;1=\sum_{\phi ,\psi }\frac{q_{\phi ,\psi }}{\exp\left(1-\lambda_{0}\right)}\exp\left(\sum_{j=1}^{m}\lambda_{j}\Gamma_{j}\left(\phi ,\psi \right)\right), \end{array}$$

the partition function is obtained as

(22) $$\begin{array}{c} \;\exp\left(1-\lambda_{0}\right)=\sum_{\phi ,\psi }q_{\phi ,\psi }\exp\left(\sum_{j=1}^{m}\lambda_{j}\Gamma_{j}\left(\phi ,\psi \right)\right)=Z. \end{array}$$

To arrive at the Lagrange Dual (10), we first rearrange the Lagrangian (17) and then substitute $p_{\phi ,\psi }$ for the MaxEnt distribution (8)/(20), making use of the normalization condition 6,

(23) $$\begin{array}{c} L=-\sum_{\phi ,\psi }p_{\phi ,\psi }\log\frac{p_{\phi ,\psi }}{q_{\phi ,\psi }}+\sum_{j=1}^{m}\lambda_{j}\sum_{\phi ,\psi }p_{\phi ,\psi }\Gamma_{j}\left(\phi ,\psi \right)\\ -\sum_{j=1}^{m}\lambda_{j}{\tilde{\Gamma }}_{j}+\lambda_{0}\left(\sum_{\phi ,\psi }p_{\phi ,\psi }-1)\right)\\ =-\sum_{\phi ,\psi }p_{\phi ,\psi }\log\frac{1}{Z}\exp\left(\sum_{j=1}^{m}\lambda_{j}\Gamma_{j}\left(\phi ,\psi \right)\right)\\ +\sum_{j=1}^{m}\lambda_{j}\sum_{\phi ,\psi }p_{\phi ,\psi }\Gamma_{j}\left(\phi ,\psi \right)-\sum_{j=1}^{m}\lambda_{j}{\tilde{\Gamma }}_{j} \end{array}\begin{array}{cccc} & = & \log Z-\sum_{\phi ,\psi }p_{\phi ,\psi }\sum_{j=1}^{m}\lambda_{j}\Gamma_{j}\left(\phi ,\psi \right) & \end{array}\begin{array}{cccc} & & +\sum_{j=1}^{m}\lambda_{j}\sum_{\phi ,\psi }p_{\phi ,\psi }\Gamma_{j}\left(\phi ,\psi \right)-\sum_{j=1}^{m}\lambda_{j}{\tilde{\Gamma }}_{j} & \end{array}\begin{array}{cccc} & = & \log Z-\sum_{j=1}^{m}\lambda_{j}{\tilde{\Gamma }}_{j}. & \end{array}$$

## Conflict of interest

The authors declare no conflict of interest.

## Supporting information

As a service to our authors and readers, this journal provides supporting information supplied by the authors. Such materials are peer reviewed and may be re‐organized for online delivery, but are not copy‐edited or typeset. Technical support issues arising from supporting information (other than missing files) should be addressed to the authors.

Supplementary

## References

[1] M. Nilges , M. Habeck , W. Rieping , C. R. Chim. 2008, 11, 356–369.

[2] A. Tantos , K.-H. Han , P. Tompa , Mol. Cell. Endocrinol. 2012, 348, 457–465. 21782886 10.1016/j.mce.2011.07.015

[3] A. Garcia-Pino , S. Balasubramanian , L. Wyns , E. Gazit , H. De Greve , R. D. Magnuson , D. Charlier , N. A. J. van Nuland , R. Loris , Cell 2010, 142, 101–111. 20603017 10.1016/j.cell.2010.05.039

[4] A. C. M. Ferreon , J. C. Ferreon , P. E. Wright , A. A. Deniz , Nature 2013, 498, 390–394. 23783631 10.1038/nature12294 PMC3718496

[5] H. N. Motlagh , J. O. Wrabl , J. Li , V. J. Hilser , Nature 2014, 508, 331–339. 24740064 10.1038/nature13001 PMC4224315

[6] P. Tompa , Trends Biochem. Sci. 2012, 37, 509–516. 22989858 10.1016/j.tibs.2012.08.004

[7] H. J. Dyson , P. E. Wright , Chem. Rev. 2004, 104, 3607–3622. 15303830 10.1021/cr030403s

[8] T. Mittag , J. D. Forman-Kay , Curr. Opin. Struct. Biol. 2007, 17, 3–14,. 17250999 10.1016/j.sbi.2007.01.009

[9] A. K. Mittermaier , L. E. Kay , Trends Biochem. Sci. 2009, 34, 601–611. 19846313 10.1016/j.tibs.2009.07.004

[10] H. Schwalbe , T. Carlomagno , M. Hennig , J. Junker , B. Reif , C. Richter , C. Griesinger , et al., Methods Enzymol. 2002, 338, 35–81. 10.1016/s0076-6879(02)38215-6 11460558

[11] B. Vögeli , L. Vugmeyster , ChemPhysChem 2019, 20, 178–196. 30110510 10.1002/cphc.201800602 PMC9206835

[12] D. Yang , L. E. Kay , J. Am. Chem. Soc. 1998, 120, 9880–9887.

[13] K. Kloiber , W. Schüler , R. Konrat , J. Biomol. NMR 2002, 22, 349–363. 12018482 10.1023/a:1014936319712

[14] C. Kauffmann , K. Kazimierczuk , T. C. Schwarz , R. Konrat , A. Zawadzka-Kazimierczuk , J. Biomol. NMR 2020, 74, 257–265. 32239382 10.1007/s10858-020-00308-y PMC7211790

[15] F. Cisnetti , K. Loth , P. Pelupessy , G. Bodenhausen , ChemPhysChem 2004, 5, 807–814. 15253308 10.1002/cphc.200301041

[16] K. Loth , P. Pelupessy , G. Bodenhausen , J. Am. Chem. Soc. 2005, 127, 6062–6068. 15839707 10.1021/ja042863o

[17] L. Vugmeyster , C. J. McKnight , Biophys. J. 2008, 95, 5941–5950. 18820237 10.1529/biophysj.108.134320 PMC2599813

[18] R. B. Fenwick , S. Esteban-Martín , B. Richter , D. Lee , K. F. A. Walter , D. Milovanovic , S. Becker , N. A. Lakomek , C. Griesinger , X. Salvatella , J. Am. Chem. Soc. 2011, 133, 10336– 10339. 21634390 10.1021/ja200461n PMC3686050

[19] R. B. Fenwick , C. D. Schwieters , B. Vögeli , J. Am. Chem. Soc. 2016, 138, 8412–8421. 27331619 10.1021/jacs.6b01447 PMC5055379

[20] T. M. Sabo , V. Gapsys , K. F. A. Walter , R. B. Fenwick , S. Becker , X. Salvatella , B. L. de Groot , D. Lee , C. Griesinger , Methods 2018, 138–139, 85–92. 10.1016/j.ymeth.2018.04.007 29656081

[21] G. Hummer , J. Köfinger , J. Chem. Phys. 2015, 143, 243150. 26723635 10.1063/1.4937786

[22] A. Cesari , S. Reißer , G. Bussi , Computation 2018, 6, 15.

[23] S. Bottaro , G. Bussi , S. D. Kennedy , D. H. Turner , K. Lindorff-Larsen , Sci. Adv. 2018, 4, eaar8521. 29795785 10.1126/sciadv.aar8521 PMC5959319

[24] J. Köfinger , L. S. Stelzl , K. Reuter , C. Allande , K. Reichel , G. Hummer , J. Chem. Theory Comput. 2019, 15, 3390–3401. 30939006 10.1021/acs.jctc.8b01231 PMC6727217

[25] S. Orioli , A. Haahr Larsen , S. Bottaro , K. Lindorff-Larsen , in Prog. Mol. Biol. Transl. Sci., Vol. 170, Elsevier, 2020, pp. 123–176. 10.1016/bs.pmbts.2019.12.006 32145944

[26] R. Rangan , M. Bonomi , G. T. Heller , A. Cesari , G. Bussi , M. Vendruscolo , J. Chem. Theory Comput. 2018, 14, 6632–6641. 30428663 10.1021/acs.jctc.8b00738

[27] B. Reif , M. Hennig , C. Griesinger , Science 1997, 276, 1230–1233. 9157875 10.1126/science.276.5316.1230

[28] D. Yang , R. Konrat , L. E. Kay , J. Am. Chem. Soc. 1997, 119, 11938–11940.

[29] P. Pelupessy , E. Chiarparin , R. Ghose , G. Bodenhausen , J. Biomol. NMR 1999, 14, 277–280. 10.1023/a:1008383205836 10383199

[30] K. Kloiber , R. Konrat , J. Biomol. NMR 2000, 17, 265–268. 10959633 10.1023/a:1008393903160

[31] N. R. Skrynnikov , R. Konrat , D. R. Muhandiram , L. E. Kay , J. Am. Chem. Soc. 2000, 122, 7059–7071.

[32] K. Kloiber , R. Konrat , J. Am. Chem. Soc. 2000, 122, 12033–12034.

[33] E. Chiarparin , P. Pelupessy , R. Ghose , G. Bodenhausen , J. Am. Chem. Soc. 2000, 122, 1758–1761.

[34] P. Pelupessy , S. Ravindranathan , G. Bodenhausen , J. Biomol. NMR 2003, 25, 265–280. 12766390 10.1023/a:1023076212536

[35] A. Kumar , R. C. R. Grace , P. K. Madhu , Nucl. Magn. Reson. 2000, 37, 191–319.

[36] G. Lipari , A. Szabo , J. Am. Chem. Soc. 1982, 104, 4546–4559.

[37] B. Vögeli , J. Chem. Phys. 2010, 133, 014501. 20614970 10.1063/1.3454734

[38] E. T. Jaynes , Phys. Rev. 1957, 108, 171.

[39] I. Csiszár , Entropy 2008, 10, 261–273.

[40] J. Shore , R. Johnson , IEEE Trans. Inf. Theory 1980, 26, 26–37.

[41] J. Skilling , in Maximum-Entropy and Bayesian Methods in Science and Engineering, Springer, 1988, pp. 173–187.

[42] J. B. Paris , A. Vencovská , Int. J. Approx. Reasoning 1990, 4, 183–223.

[43] I. Csiszar , The annals of statistics 1991, 19, 2032–2066.

[44] I. Csiszar , in Maximum entropy and Bayesian methods, Springer, 1996, pp. 35–50.

[45] K. H. Knuth , J. Skilling , Axioms 2012, 1, 38–73.

[46] P. M. Williams , British J. Philosophy Science 1980, 31, 131–144.

[47] A. Caticha , AIP Conf. Proc. 2004, 707, 75–96.

[48] A. Caticha , A. Giffin , AIP Conf. Proc. 2006, 872, 31–42.

[49] Z. Wu , G. N. Phillips Jr , R. Tapia , Y. Zhang , SIAM Review 2001, 43, 623–642.

[50] G. Bricogne , Acta Crystallogr. Sect. A 1984, 40, 410–445.

[51] G Bricogne , Acta Crystallogr. Sect. D 1993, 49, 37–60. 15299544 10.1107/S0907444992010400

[52] E. T. Jaynes, *Probability theory: The logic of science*, Cambridge university press, **2003**.

[53] C. E. Shannon , Bell Syst. Tech. J. 1948, 27, 379–423.

[54] S. Kullback , R. A. Leibler , Ann. Math. Stat. 1951, 22, 79–86.

[55] A. Charnes , W. Wager Cooper , Atti Accad. Naz. Lincei, Cl. Sci. Fis., Mat. Nat., Rend. 1975, 58, 568–576.

[56] A. Charnes , W. Wager Cooper , L Seiford , Math. Operationsforschung Statistik. Series Optimization 1978, 9, 21–29.

[57] Y. Alhassid , N. Agmon , R. D. Levine , Chem. Phys. Lett. 1978, 53, 22–26.

[58] W. Rieping , M. Habeck , M. Nilges , Science 2005, 309, 303–306. 16002620 10.1126/science.1110428

[59] C. K. Fisher , A. Huang , C. M. Stultz , J. Am. Chem. Soc. 2010, 132, 14919–14927. 20925316 10.1021/ja105832g PMC2956375

[60] S. Olsson , J. Frellsen , W. Boomsma , K. V. Mardia , T. Hamelryck , PLoS One 2013, 8, e79439. 24244505 10.1371/journal.pone.0079439 PMC3820694

[61] K. A. Beauchamp , V. S. Pande , R. Das , Biophys. J. 2014, 106, 1381–1390. 24655513 10.1016/j.bpj.2014.02.009 PMC3984982

[62] B. Różycki , Y. C. Kim , G. Hummer , Structure 2011, 19, 109–116. 21220121 10.1016/j.str.2010.10.006 PMC3032427

[63] A. B. Mantsyzov , A. S. Maltsev , J. Ying , Y. Shen , G. Hummer , A. Bax , Protein Sci. 2014, 23, 1275–1290. 24976112 10.1002/pro.2511 PMC4243998

[64] A. B. Mantsyzov , Y. Shen , J. H. Lee , G. Hummer , A. Bax , J. Biomol. NMR 2015, 63, 85–95. 26219516 10.1007/s10858-015-9971-2 PMC4577467

[65] D. L. Phillips , J. ACM (JACM) 1962, 9, 84–97.

[66] A. N. Tikhonov , Soviet Math. 1963, 4, 1035–1038.

[67] A. E. Hoerl , R. W. Kennard , Technomet 1970, 12, 55–67.

[68] A. E. Hoerl , R. W. Kennard , Technomet 1970, 12, 69–82.

[69] A. D. White , G. A. Voth , J. Chem. Theory Comput. 2014, 10, 3023–3030. 26588273 10.1021/ct500320c

[70] A. Cesari , A. Gil-Ley , G. Bussi , J. Chem. Theory Comput. 2016, 12, 6192–6200. 27951677 10.1021/acs.jctc.6b00944

[71] D. B. Amirkulova , A. D. White , Mol. Simul. 2019, 45, 1285–1294.

[72] K. Miller , SIAM J. Math. Analysis 1970, 1, 52–74.

[73] P. C. Hansen , SIAM Rev. 1992, 34, 561–580.

[74] D. W. Marquardt , J. Soc. Industrial Appl. Math. 1963, 11, 431–441.

[75] S. F. Gull , G. J. Daniell , Nature 1978, 272, 686–690.

[76] A. Golan , G. Judge , J. M. Perloff , J. Am. Stat. Assoc. 1996, 91, 841–853.

[77] S. F. Chen, R. Rosenfeld. A gaussian prior for smoothing maximum entropy models. Technical report, Carnegie-Mellon Univ Pittsburgh PA School of Computer Science, **1999**.

[78] S. F. Chen , R. Rosenfeld , IEEE Trans. Speech Audio Proc. 2000, 8, 37–50.

[79] M. Dudík , S. J. Phillips , R. E. Schapire , in International Conference on Computational Learning Theory, pages 472–486. Springer, 2004.

[80] M. Dudík , S. J. Phillips , R. E. Schapire , J. Mach. Learn. Res. 2007, 8, 1217–1260.

[81] O. F. Lange , N.-A. Lakomek , C. Farés , G. F. Schröder , K. F. A. Walter , S. Becker , J. Meiler , H. Grubmüller , C. Griesinger , B. L. de Groot , Science 2008, 320, 1471–1475. 18556554 10.1126/science.1157092

[82] B. Vögeli , J. Biomol. NMR 2017, 67, 211–232. 28286915 10.1007/s10858-017-0098-5

[83] P. A. Karplus , Protein Sci. 1996, 5, 1406–1420. 8819173 10.1002/pro.5560050719 PMC2143451

[84] F. Marinelli , J. D. Faraldo-Gómez , Biophys. J. 2015, 108, 2779–2782. 26083917 10.1016/j.bpj.2015.05.024 PMC4472218

[85] A. D. White , J. F. Dama , G. A. Voth , J. Chem. Theory Comput. 2015, 11, 2451–2460. 26575545 10.1021/acs.jctc.5b00178

[86] M. D. Hanwell , D. E. Curtis , D. C. Lonie , T. Vandermeersch , E. Zurek , G. R. Hutchison , J. Cheminform. 2012, 4, 17. 22889332 10.1186/1758-2946-4-17 PMC3542060

[87] R. A. Engh, R. Huber, *Structure quality and target parameters*, chapter 18.3, pages 382–392. American Cancer Society, **2006**.

[88] F. A. Momany , R. F. McGuire , A. W. Burgess , H. A. Scheraga , J. Phys. Chem. 1975, 79, 2361–2381.

[89] Q. Teng , M. Iqbal , T. A. Cross , J. Am. Chem. Soc. 1992, 114, 5312–5321.

[90] G. Cornilescu , J. L. Marquardt , M. Ottiger , A. Bax , J. Am. Chem. Soc. 1998, 120, 6836–6837.

[91] D. M. Schneider , M. J. Dellwo , A. J. Wand , Biochemistry 1992, 31, 3645–3652. 1314645 10.1021/bi00129a013

[92] A. B. Mantsyzov, Y. Shen, J. H. Lee, G. Hummer, A. Bax, *MERA: Maximum Entropy Ramachandran map Analysis from NMR data*, **2015** https://spin.niddk.nih.gov/bax/software/MERA (accessed August 26, 2020).

[93] E. Jones, T. Oliphant, P. Peterson, SciPy: Open source scientific tools for Python, **2001**.

[94] J. Moré , in Numerical analysis, Springer, 1978, pp. 105–116.

[95] V. Y. Orekhov, V. Jaravine, M. Mayzel, K. Kazimierczuk, MddNMR – Reconstruction of NMR spectra from NUS signal using MDD and CS, 2004–**2020**.

[96] T. D. Goddard, D. G. Kneller. Sparky 3, University of California, San Francisco, **2002**.

