# Supplementary material for: Using Cross‐Correlated Spin Relaxation to Characterize Backbone Dihedral Angle Distributions of Flexible Protein Segments
Source: Chemphyschem. 2020 Dec 10;22(1):18–28. doi: 10.1002/cphc.202000789 (PMC7839595; doi:10.1002/cphc.202000789)
Supplement: Supplementary file 1 — Supplementary [file CPHC-22-18-s001.pdf]

ChemPhysChem

Supporting Information

Using Cross-Correlated Spin Relaxation to Characterize Backbone Dihedral Angle Distributions of Flexible Protein Segments

Clemens Kauffmann,* Anna Zawadzka-Kazimierczuk, Georg Kontaxis, and Robert Konrat*

Table S1: The model protein backbone in x,y,z-coordinates with $\phi = \psi = \omega = -180°$ used to calculate the angular dependencies of all employed CCR rates.

atom	x [Å]	y [Å]	z [Å]
C'_0	2.16413	-0.09939	-1.13575
O_0	1.63267	-0.19660	-2.24073
N_1	1.46000	0.00000	0.00000
H^N_1	1.86833	0.00844	0.91280
C^α_1	0.00000	0.00000	0.00000
H^α_1	-0.24792	0.81001	-0.53144
C^β_1	-0.53833	-1.26272	-0.67575
C'_1	-0.54830	0.12453	1.42294
O_1	0.22409	0.20798	2.37653
N_2	-1.88047	0.13714	1.56711
H^N_2	-2.52627	0.13891	0.80361

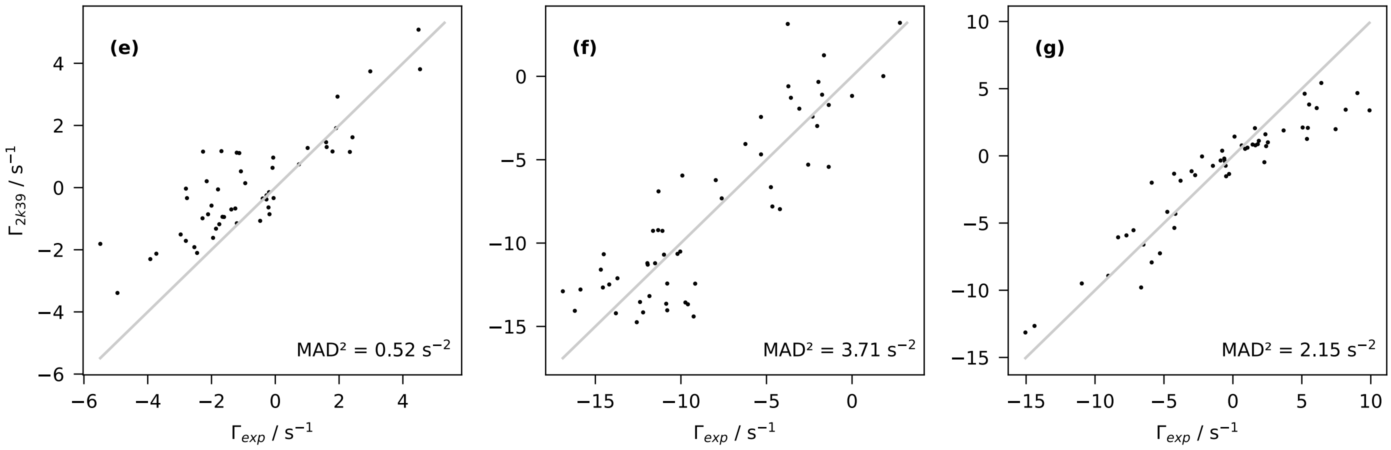

Figure S1: Comparison of CCR rates Γ_{2k39} calculated from the Lange ensemble, PDB code 2k39, and the experimentally obtained rates Γ_{exp}, (e) $\Gamma_{N_iH^N_i,C'_i}(\phi,\psi)$, (f) $\Gamma_{C^\alpha_iH^\alpha_i,N_iH^N_i}(\phi)$, (g) $\Gamma_{C^\alpha_iH^\alpha_i,C'_i}(\psi)$.

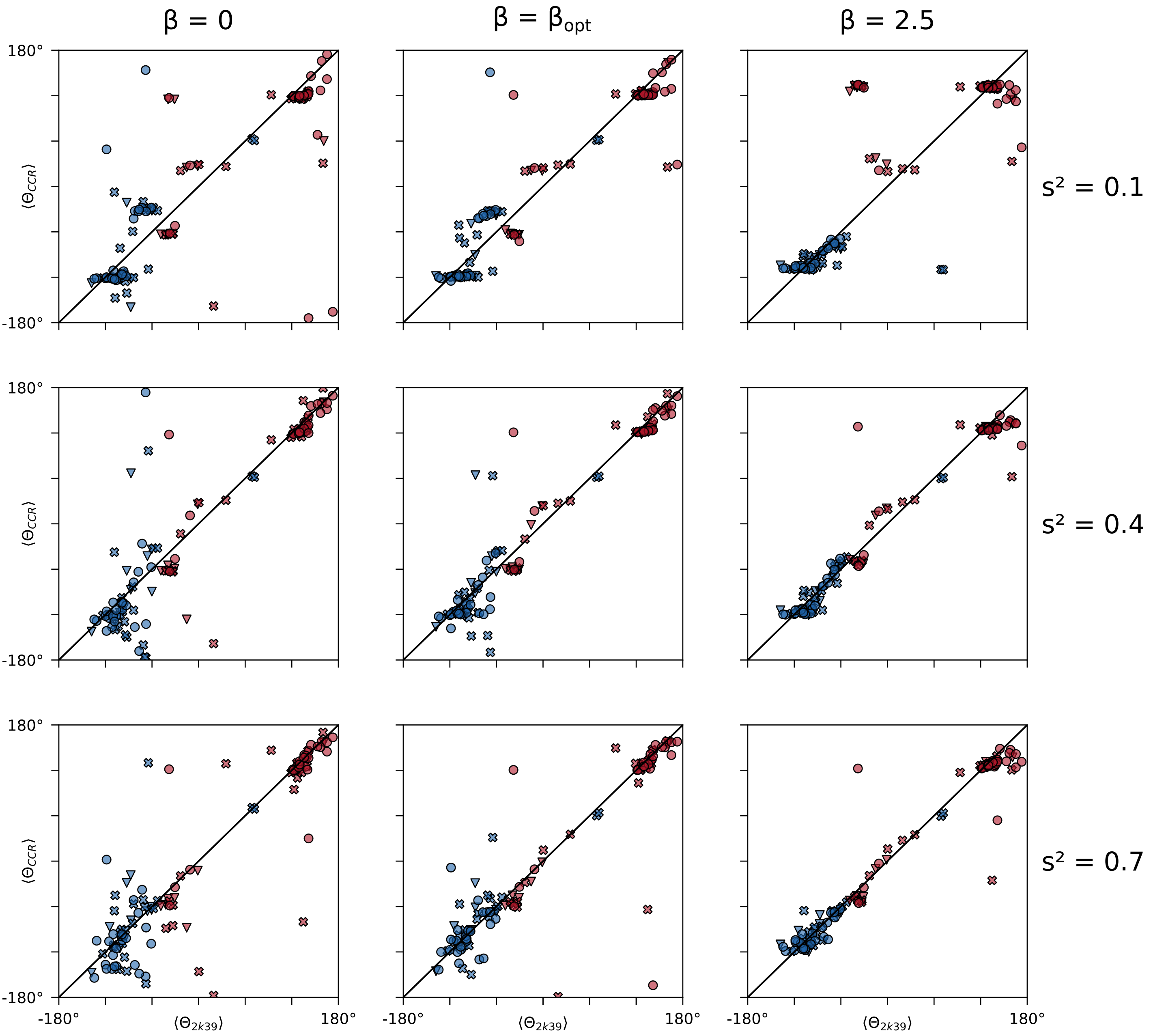

Figure S2: Comparison of average backbone dihedral angles $\langle\theta\rangle$ in Ubiquitin between the Lange ensemble, PDB code 2k39, and the CCR-derived (ϕ, ψ)-distributions obtained from Eq. (16), main text, with different S^2 (rows) and β (columns) using the random coil prior, Fig. S6. β_{opt} refers to the knee-point of the residue-specific L-curve. Different markers indicate the number of experimental CCR rates used, same as in Fig. 4, main text. Red: ψ. Blue: ϕ.

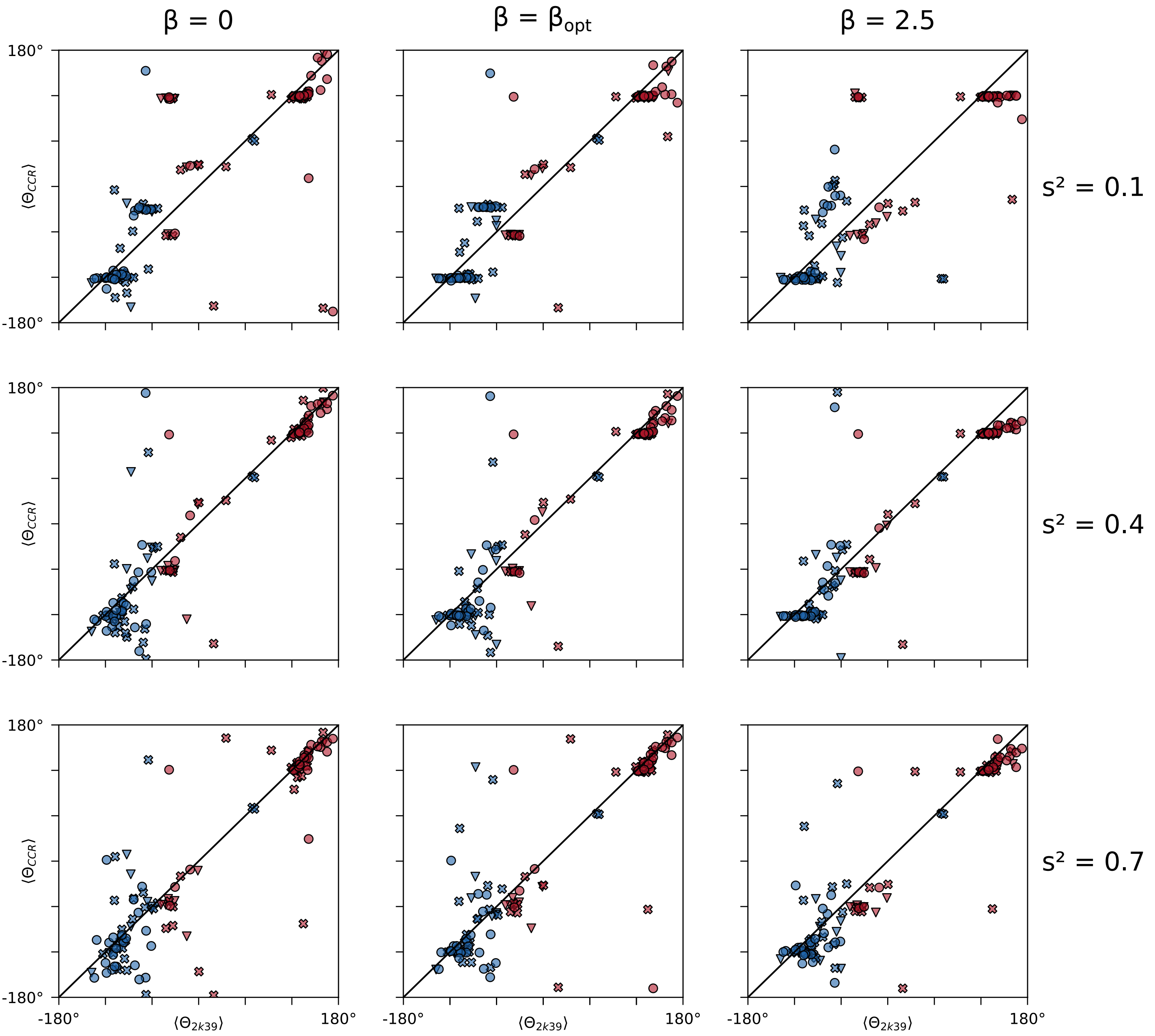

Figure S3: Comparison of average backbone dihedral angles $\langle\theta\rangle$ in Ubiquitin between the Lange ensemble, PDB code 2k39, and the CCR-derived (ϕ, ψ)-distributions obtained from Eq. (16), main text, with different S^2 (rows) and β (columns) using the uniform prior. β_{opt} refers to the knee-point of the residue-specific L-curve. Different markers indicate the number of experimental CCR rates used, same as in Fig. 4, main text. Red: ψ. Blue: ϕ.

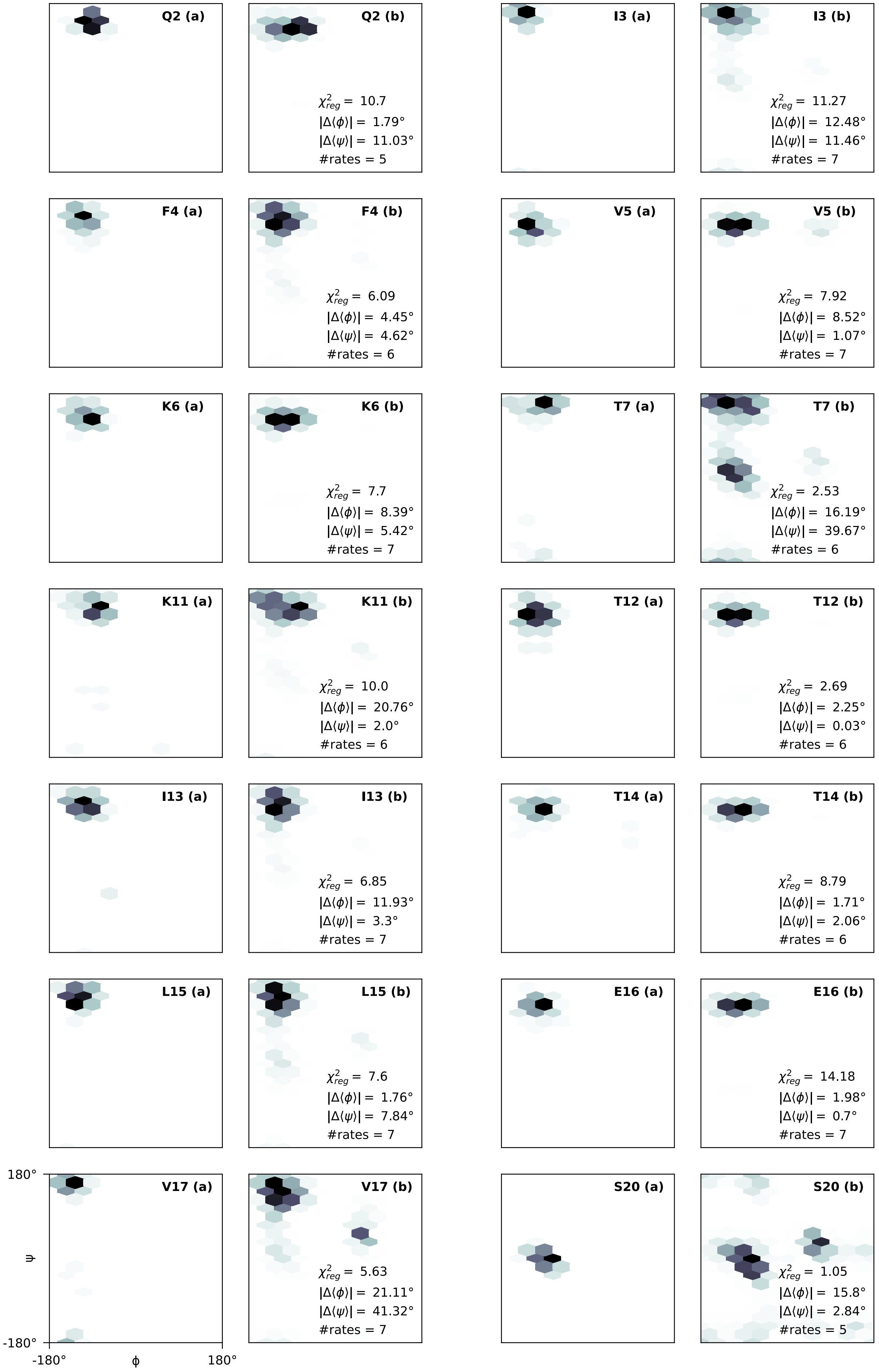
Q2 (a)
Q2 (b)
χ^2_{reg} = 10.7
$|\Delta\langle\phi\rangle|$ = 1.79°
$|\Delta\langle\psi\rangle|$ = 11.03°
#rates = 5
I3 (a)
I3 (b)
χ^2_{reg} = 11.27
$|\Delta\langle\phi\rangle|$ = 12.48°
$|\Delta\langle\psi\rangle|$ = 11.46°
#rates = 7
F4 (a)
F4 (b)
χ^2_{reg} = 6.09
$|\Delta\langle\phi\rangle|$ = 4.45°
$|\Delta\langle\psi\rangle|$ = 4.62°
#rates = 6
V5 (a)
V5 (b)
χ^2_{reg} = 7.92
$|\Delta\langle\phi\rangle|$ = 8.52°
$|\Delta\langle\psi\rangle|$ = 1.07°
#rates = 7
K6 (a)
K6 (b)
χ^2_{reg} = 7.7
$|\Delta\langle\phi\rangle|$ = 8.39°
$|\Delta\langle\psi\rangle|$ = 5.42°
#rates = 7
T7 (a)
T7 (b)
χ^2_{reg} = 2.53
$|\Delta\langle\phi\rangle|$ = 16.19°
$|\Delta\langle\psi\rangle|$ = 39.67°
#rates = 6
K11 (a)
K11 (b)
χ^2_{reg} = 10.0
$|\Delta\langle\phi\rangle|$ = 20.76°
$|\Delta\langle\psi\rangle|$ = 2.0°
#rates = 6
T12 (a)
T12 (b)
χ^2_{reg} = 2.69
$|\Delta\langle\phi\rangle|$ = 2.25°
$|\Delta\langle\psi\rangle|$ = 0.03°
#rates = 6
I13 (a)
I13 (b)
χ^2_{reg} = 6.85
$|\Delta\langle\phi\rangle|$ = 11.93°
$|\Delta\langle\psi\rangle|$ = 3.3°
#rates = 7
T14 (a)
T14 (b)
χ^2_{reg} = 8.79
$|\Delta\langle\phi\rangle|$ = 1.71°
$|\Delta\langle\psi\rangle|$ = 2.06°
#rates = 6
L15 (a)
L15 (b)
χ^2_{reg} = 7.6
$|\Delta\langle\phi\rangle|$ = 1.76°
$|\Delta\langle\psi\rangle|$ = 7.84°
#rates = 7
E16 (a)
E16 (b)
χ^2_{reg} = 14.18
$|\Delta\langle\phi\rangle|$ = 1.98°
$|\Delta\langle\psi\rangle|$ = 0.7°
#rates = 7
180°
ψ
-180°
-180°
ϕ
180°
V17 (a)
V17 (b)
χ^2_{reg} = 5.63
$|\Delta\langle\phi\rangle|$ = 21.11°
$|\Delta\langle\psi\rangle|$ = 41.32°
#rates = 7
S20 (a)
S20 (b)
χ^2_{reg} = 1.05
$|\Delta\langle\phi\rangle|$ = 15.8°
$|\Delta\langle\psi\rangle|$ = 2.84°
#rates = 5

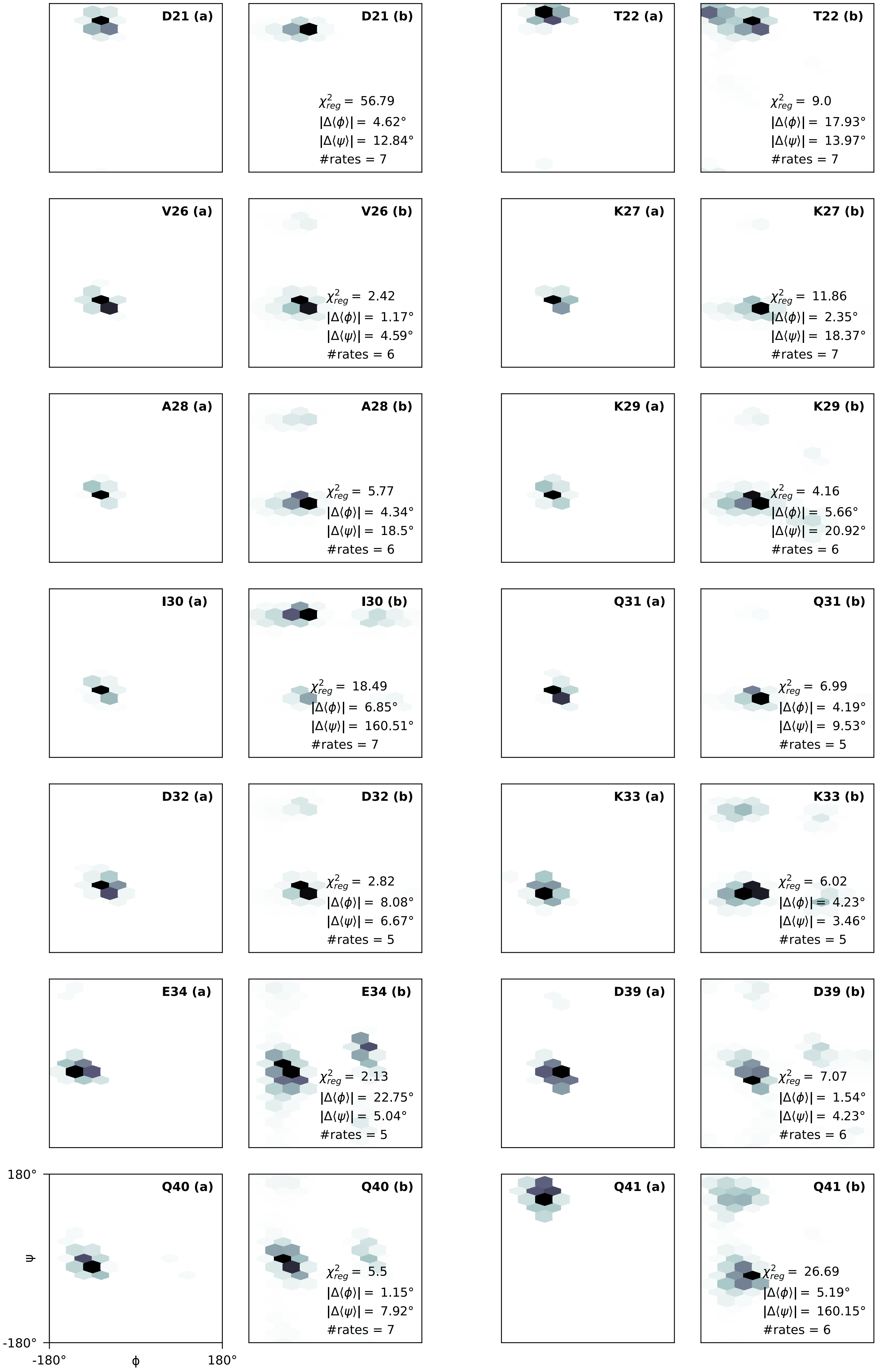
D21 (a)
D21 (b)
χ^2_{reg} = 56.79
|Δ⟨ϕ⟩| = 4.62°
|Δ⟨ψ⟩| = 12.84°
#rates = 7
T22 (a)
T22 (b)
χ^2_{reg} = 9.0
|Δ⟨ϕ⟩| = 17.93°
|Δ⟨ψ⟩| = 13.97°
#rates = 7
V26 (a)
V26 (b)
χ^2_{reg} = 2.42
|Δ⟨ϕ⟩| = 1.17°
|Δ⟨ψ⟩| = 4.59°
#rates = 6
K27 (a)
K27 (b)
χ^2_{reg} = 11.86
|Δ⟨ϕ⟩| = 2.35°
|Δ⟨ψ⟩| = 18.37°
#rates = 7
A28 (a)
A28 (b)
χ^2_{reg} = 5.77
|Δ⟨ϕ⟩| = 4.34°
|Δ⟨ψ⟩| = 18.5°
#rates = 6
K29 (a)
K29 (b)
χ^2_{reg} = 4.16
|Δ⟨ϕ⟩| = 5.66°
|Δ⟨ψ⟩| = 20.92°
#rates = 6
I30 (a)
I30 (b)
χ^2_{reg} = 18.49
|Δ⟨ϕ⟩| = 6.85°
|Δ⟨ψ⟩| = 160.51°
#rates = 7
Q31 (a)
Q31 (b)
χ^2_{reg} = 6.99
|Δ⟨ϕ⟩| = 4.19°
|Δ⟨ψ⟩| = 9.53°
#rates = 5
D32 (a)
D32 (b)
χ^2_{reg} = 2.82
|Δ⟨ϕ⟩| = 8.08°
|Δ⟨ψ⟩| = 6.67°
#rates = 5
K33 (a)
K33 (b)
χ^2_{reg} = 6.02
|Δ⟨ϕ⟩| = 4.23°
|Δ⟨ψ⟩| = 3.46°
#rates = 5
E34 (a)
E34 (b)
χ^2_{reg} = 2.13
|Δ⟨ϕ⟩| = 22.75°
|Δ⟨ψ⟩| = 5.04°
#rates = 5
D39 (a)
D39 (b)
χ^2_{reg} = 7.07
|Δ⟨ϕ⟩| = 1.54°
|Δ⟨ψ⟩| = 4.23°
#rates = 6
Q40 (a)
Q40 (b)
χ^2_{reg} = 5.5
|Δ⟨ϕ⟩| = 1.15°
|Δ⟨ψ⟩| = 7.92°
#rates = 7
Q41 (a)
Q41 (b)
χ^2_{reg} = 26.69
|Δ⟨ϕ⟩| = 5.19°
|Δ⟨ψ⟩| = 160.15°
#rates = 6
180°
ψ
-180°
-180°
ϕ
180°

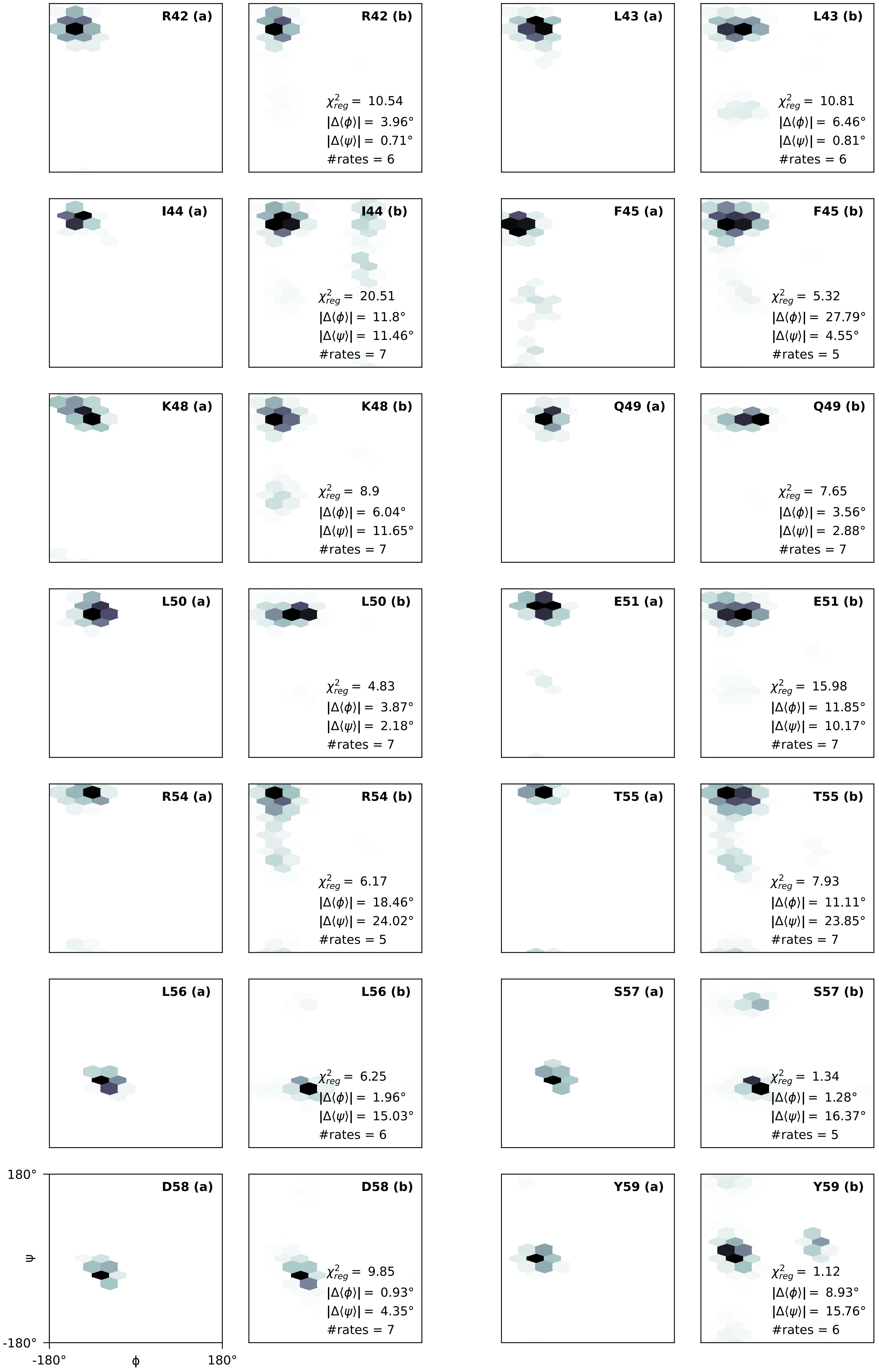
R42 (a)
R42 (b)
χ^2_{reg} = 10.54
|Δ⟨φ⟩| = 3.96°
|Δ⟨ψ⟩| = 0.71°
#rates = 6
L43 (a)
L43 (b)
χ^2_{reg} = 10.81
|Δ⟨φ⟩| = 6.46°
|Δ⟨ψ⟩| = 0.81°
#rates = 6
I44 (a)
I44 (b)
χ^2_{reg} = 20.51
|Δ⟨φ⟩| = 11.8°
|Δ⟨ψ⟩| = 11.46°
#rates = 7
F45 (a)
F45 (b)
χ^2_{reg} = 5.32
|Δ⟨φ⟩| = 27.79°
|Δ⟨ψ⟩| = 4.55°
#rates = 5
K48 (a)
K48 (b)
χ^2_{reg} = 8.9
|Δ⟨φ⟩| = 6.04°
|Δ⟨ψ⟩| = 11.65°
#rates = 7
Q49 (a)
Q49 (b)
χ^2_{reg} = 7.65
|Δ⟨φ⟩| = 3.56°
|Δ⟨ψ⟩| = 2.88°
#rates = 7
L50 (a)
L50 (b)
χ^2_{reg} = 4.83
|Δ⟨φ⟩| = 3.87°
|Δ⟨ψ⟩| = 2.18°
#rates = 7
E51 (a)
E51 (b)
χ^2_{reg} = 15.98
|Δ⟨φ⟩| = 11.85°
|Δ⟨ψ⟩| = 10.17°
#rates = 7
R54 (a)
R54 (b)
χ^2_{reg} = 6.17
|Δ⟨φ⟩| = 18.46°
|Δ⟨ψ⟩| = 24.02°
#rates = 5
T55 (a)
T55 (b)
χ^2_{reg} = 7.93
|Δ⟨φ⟩| = 11.11°
|Δ⟨ψ⟩| = 23.85°
#rates = 7
L56 (a)
L56 (b)
χ^2_{reg} = 6.25
|Δ⟨φ⟩| = 1.96°
|Δ⟨ψ⟩| = 15.03°
#rates = 6
S57 (a)
S57 (b)
χ^2_{reg} = 1.34
|Δ⟨φ⟩| = 1.28°
|Δ⟨ψ⟩| = 16.37°
#rates = 5
180°
ψ
-180°
-180°
ϕ
180°
D58 (a)
D58 (b)
χ^2_{reg} = 9.85
|Δ⟨φ⟩| = 0.93°
|Δ⟨ψ⟩| = 4.35°
#rates = 7
Y59 (a)
Y59 (b)
χ^2_{reg} = 1.12
|Δ⟨φ⟩| = 8.93°
|Δ⟨ψ⟩| = 15.76°
#rates = 6

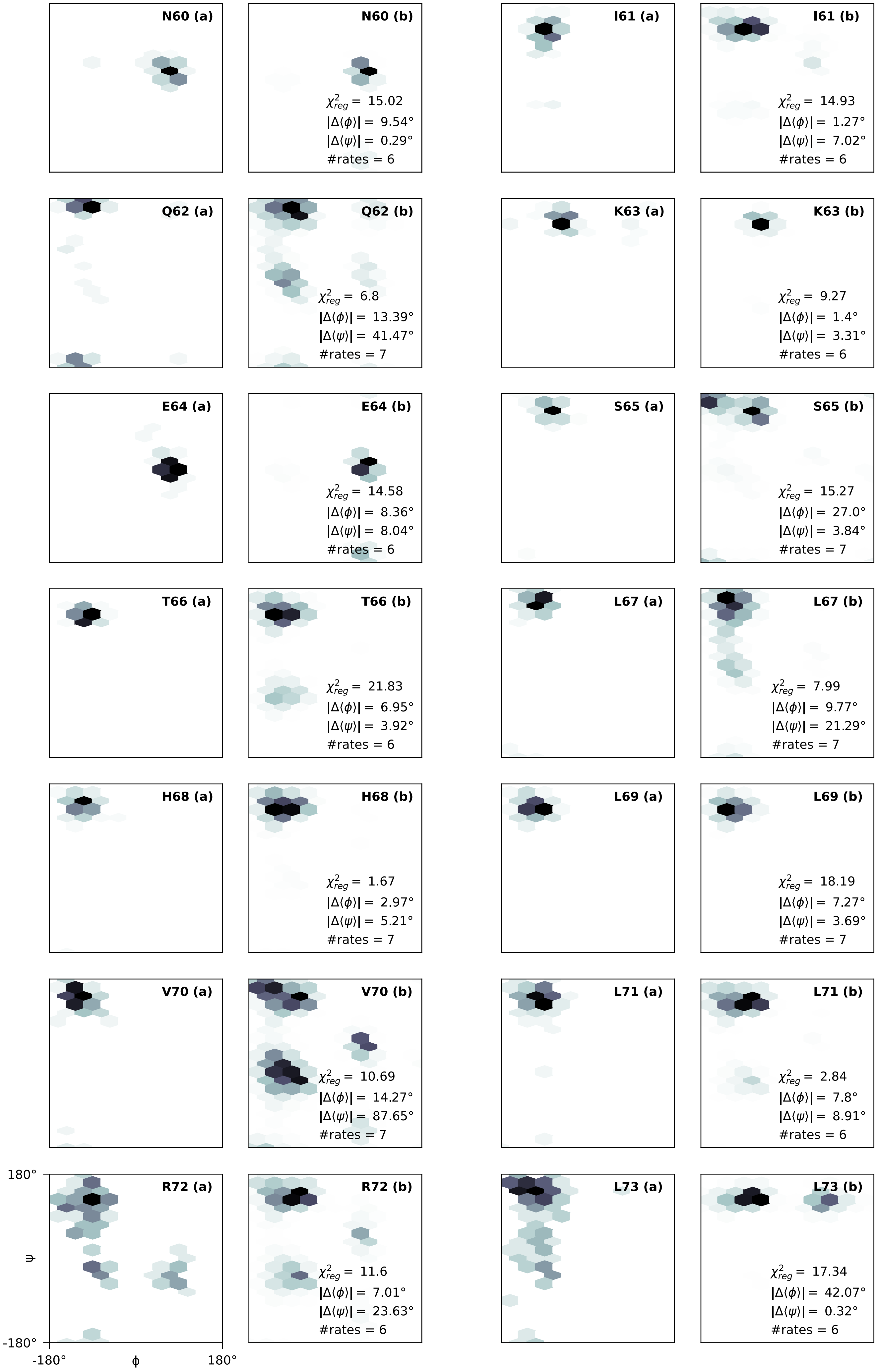
N60 (a)
N60 (b)
χ^2_{reg} = 15.02
|Δ⟨ϕ⟩| = 9.54°
|Δ⟨ψ⟩| = 0.29°
#rates = 6
I61 (a)
I61 (b)
χ^2_{reg} = 14.93
|Δ⟨ϕ⟩| = 1.27°
|Δ⟨ψ⟩| = 7.02°
#rates = 6
Q62 (a)
Q62 (b)
χ^2_{reg} = 6.8
|Δ⟨ϕ⟩| = 13.39°
|Δ⟨ψ⟩| = 41.47°
#rates = 7
K63 (a)
K63 (b)
χ^2_{reg} = 9.27
|Δ⟨ϕ⟩| = 1.4°
|Δ⟨ψ⟩| = 3.31°
#rates = 6
E64 (a)
E64 (b)
χ^2_{reg} = 14.58
|Δ⟨ϕ⟩| = 8.36°
|Δ⟨ψ⟩| = 8.04°
#rates = 6
S65 (a)
S65 (b)
χ^2_{reg} = 15.27
|Δ⟨ϕ⟩| = 27.0°
|Δ⟨ψ⟩| = 3.84°
#rates = 7
T66 (a)
T66 (b)
χ^2_{reg} = 21.83
|Δ⟨ϕ⟩| = 6.95°
|Δ⟨ψ⟩| = 3.92°
#rates = 6
L67 (a)
L67 (b)
χ^2_{reg} = 7.99
|Δ⟨ϕ⟩| = 9.77°
|Δ⟨ψ⟩| = 21.29°
#rates = 7
H68 (a)
H68 (b)
χ^2_{reg} = 1.67
|Δ⟨ϕ⟩| = 2.97°
|Δ⟨ψ⟩| = 5.21°
#rates = 7
L69 (a)
L69 (b)
χ^2_{reg} = 18.19
|Δ⟨ϕ⟩| = 7.27°
|Δ⟨ψ⟩| = 3.69°
#rates = 7
V70 (a)
V70 (b)
χ^2_{reg} = 10.69
|Δ⟨ϕ⟩| = 14.27°
|Δ⟨ψ⟩| = 87.65°
#rates = 7
L71 (a)
L71 (b)
χ^2_{reg} = 2.84
|Δ⟨ϕ⟩| = 7.8°
|Δ⟨ψ⟩| = 8.91°
#rates = 6
R72 (a)
R72 (b)
χ^2_{reg} = 11.6
|Δ⟨ϕ⟩| = 7.01°
|Δ⟨ψ⟩| = 23.63°
#rates = 6
L73 (a)
L73 (b)
χ^2_{reg} = 17.34
|Δ⟨ϕ⟩| = 42.07°
|Δ⟨ψ⟩| = 0.32°
#rates = 6
180°
ψ
-180°
-180°
ϕ
180°

Figure S4: Comparison of all 56 backbone dihedral angle distributions in Ubiquitin between the Lange ensemble, PDB code 2k39, in columns (a) and the CCR-derived fitting results in columns (b) obtained from Eq. (16), main text, with $S^2 = 0.7$ and $\beta = 2.5$. Residue type and number are indicated in the top right corner. $|\Delta\langle\phi\rangle|$ and $|\Delta\langle\psi\rangle|$ denote the absolute difference in average dihedral angles between (a) and (b), #rates is the number of CCR rates used to derive (b).

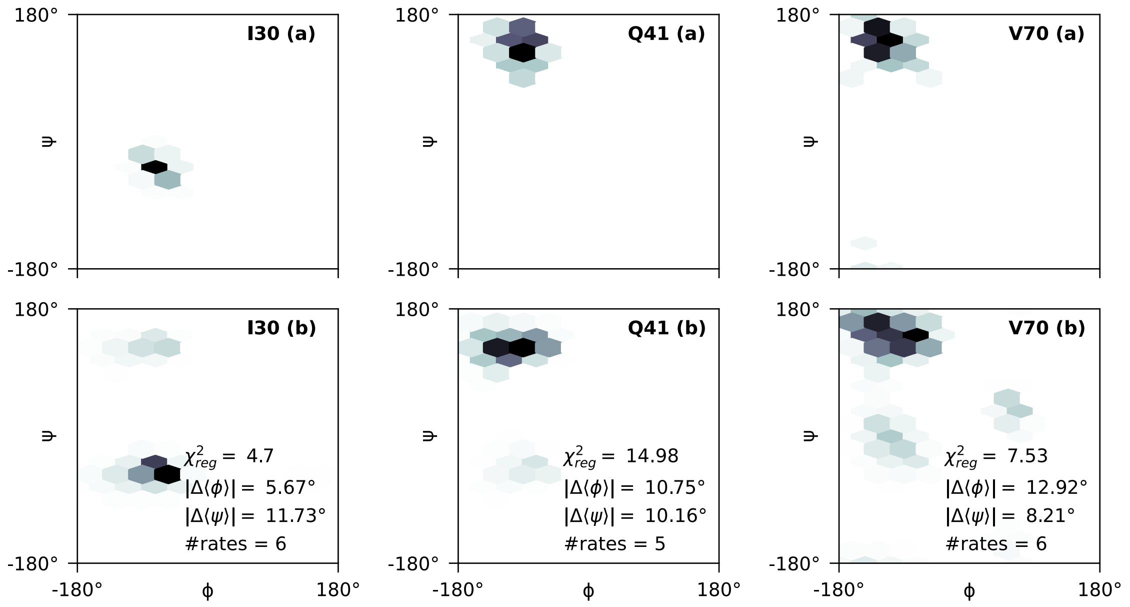

Figure S5: Comparison of three backbone dihedral angle distributions in Ubiquitin between the Lange ensemble, PDB code 2k39, in row (a) and the CCR-derived fitting results in row (b) obtained from Eq. (16), main text, with $S^2 = 0.7$ and $\beta = 2.5$ after exclusion of $\Gamma_{C_i^\alpha H_i^\alpha, N_{i+1} H_{i+1}^N}(\psi)$. Residue type and number are indicated in the top right corner. The residues correspond to the outliers highlighted in Fig. 2, main text. $|\Delta\langle\phi\rangle|$ and $|\Delta\langle\psi\rangle|$ denote the absolute difference in average dihedral angles between (a) and (b), #rates is the number of CCR rates used to derive (b).

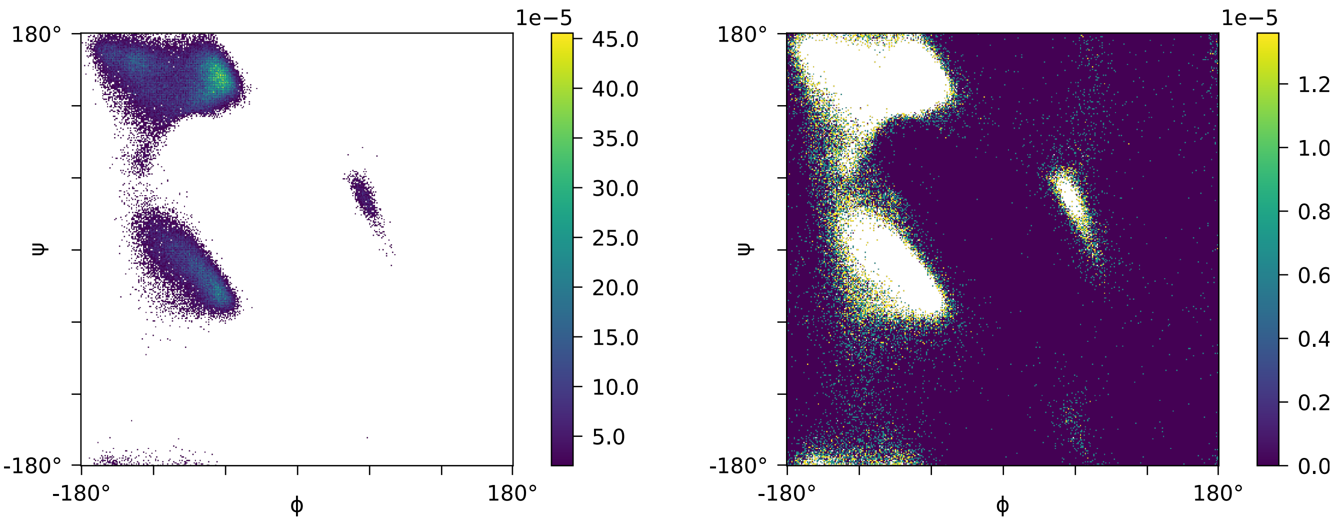

Figure S6: The random coil prior, a normalized histogram on a 360°x360° grid, as defined in the Computational Methods, main text. Left: The most populated (ϕ,ψ)-pairs accounting for 90% of the population. Right: The least populated (ϕ,ψ)-pairs accounting for 10% of the population.
